# SeqKit: A Cross-Platform and Ultrafast Toolkit for FASTA/Q File Manipulation

**DOI:** 10.1371/journal.pone.0163962

**Published:** 2016-10-05

**Authors:** Wei Shen, Shuai Le, Yan Li, Fuquan Hu

**Affiliations:** 1 Department of Microbiology, College of Basic Medical Sciences, Third Military Medical University, 30# Gaotanyan St., Shapingba District, Chongqing, China; 2 Medical Research Center, Southwest hospital, Third Military Medical University, 29# Gaotanyan St., Shapingba District, Chongqing, China; Tianjin University, CHINA

## Abstract

FASTA and FASTQ are basic and ubiquitous formats for storing nucleotide and protein sequences. Common manipulations of FASTA/Q file include converting, searching, filtering, deduplication, splitting, shuffling, and sampling. Existing tools only implement some of these manipulations, and not particularly efficiently, and some are only available for certain operating systems. Furthermore, the complicated installation process of required packages and running environments can render these programs less user friendly. This paper describes a cross-platform ultrafast comprehensive toolkit for FASTA/Q processing. SeqKit provides executable binary files for all major operating systems, including Windows, Linux, and Mac OSX, and can be directly used without any dependencies or pre-configurations. SeqKit demonstrates competitive performance in execution time and memory usage compared to similar tools. The efficiency and usability of SeqKit enable researchers to rapidly accomplish common FASTA/Q file manipulations. SeqKit is open source and available on Github at https://github.com/shenwei356/seqkit.

## Introduction

FASTA and FASTQ are basic and ubiquitous text-based formats for storing nucleotide and protein sequences. FASTA was introduced first in FASTA software [1], and FASTQ was originally developed at the Wellcome Trust Sanger Institute [2]. Common manipulations of FASTA/Q files include converting, cleaning, searching, filtering, deduplication, splitting, shuffling, and sampling. The simplicity of the FASTA/Q formats makes them easy to be parsed and manipulated with programming languages like Python and Perl. However, researchers, especially beginners, repeatedly write scripts for common purposes such as extracting sequences by using an identifiers (IDs) list file. Most of these scripts are not well organized or documented and are not reusable by other researchers. Many tools are available for the manipulation of FASTA/Q files, including fasta_utilities [3], fastx_toolkit [4], pyfaidx [5], seqmagick [6] and seqtk [7]. However, most of these programs implement only some of the above functions necessary for common manipulation and are not efficient for large files. Moreover, some tools require dependencies or running environments for installation or are only available for specific operating systems, which render them less user friendly. With the increasing number of sequences being produced, processing efficiency has become critical. Here, we introduced SeqKit toolkit to address the need for efficient and facile manipulations of FASTA/Q files.

## Method

In this work, we present a novel FASTA/Q command-line toolkit, SeqKit, which is implemented in the Go programming language, which makes it available for most popular operating systems including Windows, Linux, Mac OS X and FreeBSD. SeqKit is lightweight and can be used out-of-the-box without any dependencies or configurations, which makes it user friendly.

### Program organization

The SeqKit toolkit adopts the structure of “command subcommand”, i.e., users access functions of SeqKit from single entrance, “seqkit,” and specify a detailed function with subcommand name. Many subcommands share similar options (called flags in SeqKit), so these options are refactored as global options/flags. This structure benefits both potential developers and users learning the functionality of SeqKit.

SeqKit consists of nineteen subcommands (Table 1) that provide completely independent functions. All subcommands support plain or gzip-compressed inputs and outputs from either standard streams or local files. Therefore, SeqKit can be easily combined in a command-line pipe to accomplish complex manipulations.

**Table 1:** Subcommands of SeqKit toolkit.

Categories	Subcommands	Description
Basic operations	seq	Validating and transforming sequences
	subseq	Getting subsequences by region/GTF/BED
	sliding	Sliding sequences
	stat	Simple statistics
	faidx	Creating FASTA index files
Format conversion	fx2tab	Converting FASTA/Q to tabular format with extra information
	tab2fx	Converting tabular format to FASTA/Q format
	fq2fa	Converting FASTQ format to FASTA
Searching	grep	Searching sequences by patterns/IDs/motifs
	locate	Locating subsequences/motifs
Set operations	rmdup	Removing duplicated sequences by ID/name/seq
	common	Finding common sequences of multiple files by ID/name/seq
	split	Splitting sequences into files by ID/seq region/size/parts
	sample	Sampling sequences by number or proportion
	head	Printing the first N FASTA/Q records
Edit	replace	Editing name/sequence by regular expression
	rename	Renaming duplicated IDs
Ordering	shuffle	Shuffling sequences
	sort	Sorting sequences by ID/name/sequence/length

### FASTA/Q format parsing

**Fig 1:**
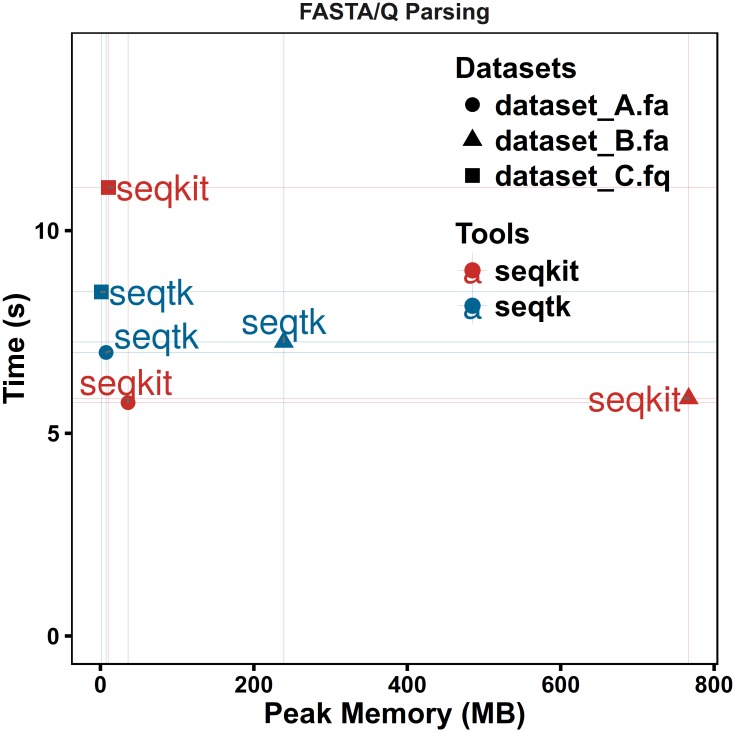
Performance comparison for FASTA/Q file parsing. Dataset A consists of 67,748 DNA sequences with average length of 41 Kb; dataset B is the human genome with 24 chromosomes, one mitochondrial sequence and 169 scaffolds and dataset C contains 9,186,045 Illumina SE reads. All tests were repeated five times, and the average time or memory usage was computed. See supplementary data for details of test data and commands.

In most cases, file I/O (input and output) is the performance bottleneck of sequence manipulation tools. SeqKit uses the self-implemented lightweight and high-performance bioinformatics package bio [8] for FASTA/Q parsing, which exhibits high performance similar to the widely used klib (kseq.h) [9] (Fig 1). SeqKit seamlessly supports both FASTA and FASTQ formats, and file type is automatically detected. All subcommands, with the exception of "faidx", can handle both formats. The two-pass mode of some commands (i.e., "subseq, "split", "sort" and "shuffle"), which utilize a FASTA index to improve processing performance for large files, only supports the FASTA format. When input files are plain or gzip-compressed FASTA files, a FASTA index would be optionally used for rapid access of sequences and to reduce memory usage. To restore the original FASTA header information, SeqKit uses a full sequence header as the sequence identifier (ID). Therefore, the FASTA index file (".seqkit.fa") created by SeqKit is slightly different from the ".fai" file created by SAMtools [10]. SeqKit also supports custom IDs using regular expressions, which allows users to customize their experience.

Sequence type (DNA/RNA/Protein) is automatically detected by the leading subsequences of the first record. User can also specify sequence type to validate letters of sequences using subcommand “seq”.

### Performance optimization

To utilize the multi-CPU environment of modern computers, most CPU-intensive processes are parallelized by multiple Goroutines in the Go programming language, which are similar to, but lighter weight than, threads. Parallelized processes include 1) validation of sequence letters; 2) computation of reverse complementary nucleotide sequences for long sequences; 3) parsing pattern and GTF/BED files; and 4) converting tabular format to FASTA/Q format.

To improve processing efficiency, SeqKit uses some customized data structures and algorithms. For example, the sequence parsing algorithm uses a custom buffered file reading algorithm instead of the Go standard library “bufio”, which is not memory-efficient for large sequences. This change increased the speed and significantly lowered the memory usage. Additionally, the computation of the reverse complementary sequence utilizes map in Go (also called hash table or dictionary in some other programming language) and is usually used to store the mapping relations of nucleotide bases and their complementary bases. However, the built-in map data structure of Go is inefficient because the key and value data types are bytes, which is actually uint8 (unsigned 8-bit integer). Instead, we use the slice data structure (similar to array in Perl and list in Python) to store complementary sequences with the ASCII code of the byte as the indexing key (Algorithm 1). This algorithm resulted in a ~20× speedup relative to the strategy of map (Table A in S1 File). The memory usage is also very low since the size of base alphabet is limited.

**Algorithm 1:** Fast reverse complementary sequence

**Function 1:** byte2int(*b*)

**Input:** Byte *b*

**return** ASCII value of byte *b*

**Function 2:** ReverseComplementarySeq(*A*, *S*)

**Input:** An array of bytes containing alphabet letters: *A* and an array of bytes: *S*

**Output:** Reverse complementary sequence of *S*: *RC*

Step 1: Creating mapping array *L*

*L* ← Initializing an array of 256 bytes with value of null characters ('\0')

**for** *b* **in** *A* **do**

 *L*[byte2int(*b*)] ← Complementary base of *b*

**end**

Step 2: Computing reverse complementary sequence

*RC* ← Reverse array of *S*

**for** *i* = 1 to (length of *S*) **do**

 *b* ← *S*[*i*]

 **if** byte2int(*b*) > 256 or *L*[byte2int(*b*)] = '\0'

  raise error

 **else**

  *RC*[*i*] ← *L*[byte2int(*b*)]

 **end**

**end**

**return** *RC*

Most subcommands of SeqKit do not load all FASTA/Q records in to memory. Some manipulations, such as removing duplicate sequences by sequence content, do need to store whole sequences in memory. SeqKit uses a MD5 digest to represent sequence content, which greatly reduces memory usage. Some subcommands can either read all records in memory, but others, including "sample", "split", "shuffle" and "sort", read the files twice in two-pass mode. This read mode uses the FASTA index for rapid access of sequences and reduces memory usage.

### Reproducibility

Reproducibility is very important in scientific tools. The results from all subcommands could be reproduced with the same dataset and arguments across different operating system. The subcommands "sample" and "shuffle" in SeqKit use random functions, so the configurability of the random seed guarantees that the results can be reproduced in different environments using the same data and commands.

## Results and Discussion

To address the needs for efficient and easy-to-use manipulations of FASTA/Q files, we present SeqKit here.

### Functions and features

With nineteen subcommands (Table 1), SeqKit provides functions covering most aspects of FASTA/Q (mainly FASTA) manipulation. SeqKit provides more comprehensive features compared to other tools (Table 2). For example, shuffling is a necessary process before splitting FASTA files for the cross-validation of machine learning algorithms. Although the GNU tool “shuf” provides a shuffling function for list files, more shell commands are needed to shuffle FASTA files. In contrast, the subcommand “shuffle” of SeqKit provides an efficient and cross-platform way to achieve this objective. Similarly, no tools provide functions for locating sequence motifs and identifying common sequences between multiple files, which are both common manipulations in research analyses.

**Table 2:** Overview FASTA/Q processing tool features.

Categories	Features	seqkit	fasta_utilities	fastx_toolkit	pyfaidx	seqmagick	seqtk
Formats supports	Multi-line FASTA	Yes	Yes	--	Yes	Yes	Yes
	FASTQ	Yes	Yes	Yes	--	Yes	Yes
	Multi-line FASTQ	Yes	Yes	--	--	Yes	Yes
	Validating sequences	Yes	--	Yes	Yes	--	--
	Supporting RNA	Yes	Yes	--	--	Yes	Yes
Functions	Searching by motifs	Yes	Yes	--	--	Yes	--
	Sampling	Yes	--	--	--	Yes	Yes
	Extracting sub-sequence	Yes	Yes	--	Yes	Yes	Yes
	Removing duplicates	Yes	--	--	--	Partly	--
	Splitting	Yes	Yes	--	Partly	--	--
	Splitting by seq	Yes	--	Yes	Yes	--	--
	Shuffling	Yes	--	--	--	--	--
	Sorting	Yes	Yes	--	--	Yes	--
	Locating motifs	Yes	--	--	--	--	--
	Common sequences	Yes	--	--	--	--	--
	Cleaning bases	Yes	Yes	Yes	Yes	--	--
	Transcription	Yes	Yes	Yes	Yes	Yes	Yes
	Translation	--	Yes	Yes	Yes	Yes	--
	Filtering by size	Indirect	Yes	--	Yes	Yes	--
	Renaming header	Yes	Yes	--	--	Yes	Yes
Other features	Cross-platform	Yes	Partly	Partly	Yes	Yes	Yes
	Reading STDIN	Yes	Yes	Yes	--	Yes	Yes
	Reading gzipped file	Yes	Yes	--	--	Yes	Yes
	Writing gzip file	Yes	--	--	--	Yes	--

For common functions also provided by other tools, SeqKit offers more practical controls with more options. For example, fasta_utilities, seqmagick and SeqKit all support searching sequences by pattern (i.e., regular expression), but SeqKit supports searching with sequence motifs containing degenerate sequences (e.g., TTSAA, the digest site of the restriction enzyme AgsI, is equal to the regular expression TT[CG]AA). SeqKit provides practical extended positioning strategies for obtaining subsequences by region (position range). In addition, for common range notation such as “1:20”, SeqKit can choose more advanced regions. For example, the last 12 bases can be identified using “-12:-1” and the whole sequence by “1:-1”. SeqKit can also extract up-stream and down-stream flanking sequences in GTF/BED files.

As a command-line tool, all subcommands of SeqKit support plain or gzip-compressed input and output from either standard stream or local files. Therefore, it can be easily combined in command-line pipes to accomplish complex manipulations. SeqKit also provides functions for converting FASTA/Q to and from tabular format, which can be conveniently manipulated with other tabular format tools including “cut”, “sort”, and “awk”.

### Computational time and memory usage

Computational efficiency and memory usage are critical with the increasing scale of sequencing data. Sequence records parsing is the main bottleneck in the manipulation of FASTA/Q files. SeqKit adopts the authors’ high-performance bioinformatics package[8], which had been successfully applied in a fast sequence processing tool [11], to parse FASTA and FASTQ files. To test the comprehensive performance on FASTA and FASTQ format parsing, three different datasets were used. Dataset A (file size: ~2.7 G) consists of 67,748 DNA sequences with average length of 41 Kb, representing large FASTA files with average sized sequences. Dataset B (file size: ~2.9 G) is the human genome with 24 chromosomes, one mitochondrial sequence and 169 scaffolds and serves as an example of large FASTA file with large sequence sizes. Dataset C (file size: ~2.2 G) contains 9,186,045 Illumina SE reads as an example of typical FASTQ files generated from next-generation sequencing. The benchmark results were compared to the widely used high-performance FASTA/Q parsing C library klib (kseq.h)[9]. SeqKit outperformed seqtk using klib in processing time on the two scales of FASTA file parsing while maintaining reasonable peak memory usage. SeqKit archived approximately 85% speed of seqtk in FASTQ file parsing (Fig 1).

SeqKit utilizes multiple CPUs to accelerate computationally intensive processes (See Method). To assess the performance improvement of multiple threads, five tests were performed with a serial number of threads (Goroutine in Go) (Figure A in S1 File). The results showed that two threads generally performed better than a single thread and that no further significant improvements were obtained with three or more threads. Therefore, the default number of threads for multi-core computers was two.

**Fig 2:**
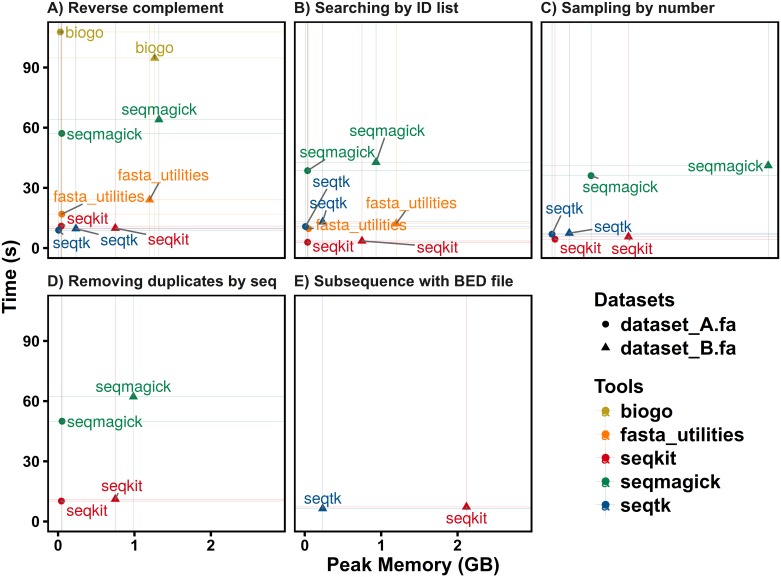
Performance comparison on five manipulations of FASTA file. Dataset A consists of 67,748 DNA sequences with average length of 41 Kb and dataset B is the human genome with 24 chromosomes, one mitochondrial sequence and 169 scaffolds. All tests were repeated three times, and the average time or memory usage was computed. See supplementary data for details of test data and commands.

To assess the comprehensive performance of SeqKit, five tests of common manipulations on FASTA/Q were performed using different tools on datasets A, B (Fig 2) and C (Figure B in S1 File). For the computation of the reverse complement sequence (Fig 2A), one of the most basic sequence manipulations, the execution time mainly depends on the efficiency of the development language, the FASTA/Q parsing and the reverse complementary sequence computing algorithm. In general, execution time inversely correlates with the speed of the programming language. Generally, seqtk written in C, and SeqKit, written in Go, required the least amount of time. The FASTA/Q parsing module of seqtk, klib [9], is highly optimized, which makes it very efficient in terms of execution time and memory usage. Interestingly, while SeqKit and the biogo package[12] were both written in Go, SeqKit was approximately 9~11 times faster than the biogo package for FASTA parsing, which indicates the efficiency of the SeqKit algorithm.

SeqKit required far less time than all other software with reasonable memory usage for searching sequences by the ID list (Fig 2B). When a FASTQ file was used (Figure B in S1 File), the performance of SeqKit did not change, while the performance of fasta_utilities and seqmagick decreased dramatically. When sampling by sequence number (Fig 2C and Figure B in S1 File), seqtk and Seqkit showed similar computational speeds. However, seqmagick used far more memory than seqtk and SeqKit because it read the whole file into memory, which may exhaust system memory when using larger datasets.

Only two software packages supported removing duplicate sequences by sequence content. SeqKit ran much faster than seqmagick and used less memory (Fig 2D and Figure B in S1 File). When getting subsequences from BED files, SeqKit and seqtk performed similarly in speed but used more memory (Fig 2E).

**Fig 3:**
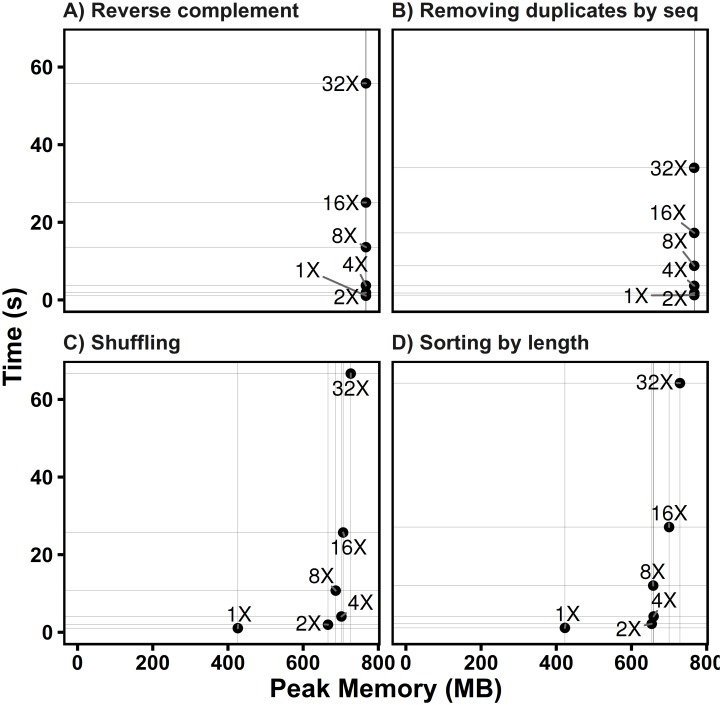
Performance of SeqKit on different data sizes. The text label represents file size relative to the human genome chromosome 1 (248,956,422 bp, file size: 241.4 Mb). All tests were repeated three times, and the average time or memory usage was computed. See supplementary data for details of test data and commands.

Since SeqKit used more memory than seqtk in all cases, we assessed the memory usage of SeqKit on different scales of data. To this end, four tests were performed on a series of files generated by repeating human chromosome 1 N times and renaming each sequence with unique identifiers. In tests of computing reverse complementary sequences and removing duplicated sequences by content, the memory usage increased with file size and stayed at approximately 780 Mb (Fig 3A and 3B). Similarly, when the FASTA index was used to access FASTA sequences for shuffling and sorting, the peak memory stayed at approximately 750 Mb. These results showed that the peak memory usage of SeqKit is determined by the length of the longest sequence record. Considering the efficiency both in time and memory, SeqKit can meet the need for efficient manipulations of large FASTA and FASTQ files with the growth of data size.

### Limitations

Although SeqKit seamlessly supports both FASTA and FASTQ format, most of the subcommands were designed to handle common manipulations. Some manipulations of FASTQ, such as trimming low-quality reads, were not included. SeqKit supports the inter-conversion of three file types, including FASTQ-FASTA and FASTA/Q-tabular format. Other next-generation sequencing formats like BAM/SAM can be converted to FASTQ using tools like bamtofastq of bedtools [13], which then can be processed by SeqKit.

## Supporting Information

S1 File SeqKit supplementary data 1. Benchmark details and results.
(PDF)

S2 File SeqKit supplementary data 2. All data supporting this article including source code, documents, executable binary files, benchmark scripts and plotting scripts.
(ZIP)
