# Supplementary material for: SeqKit: A Cross-Platform and Ultrafast Toolkit for FASTA/Q File Manipulation
Source: PLoS One. 2016 Oct 5;11(10):e0163962. doi: 10.1371/journal.pone.0163962 (PMC5051824; doi:10.1371/journal.pone.0163962)
Supplement: S1 File — Benchmark details and results. (PDF) [file pone.0163962.s001.pdf]

SeqKit: a cross-platform and ultrafast toolkit for FASTA/Q file manipulation – Supplementary data

Wei Shen[1], Shuai Le[1], Yan Li[2,*] and Fuquan Hu[1,*]

[1]Department of Microbiology, College of Basic Medical Sciences, Third Military Medical University, 30# Gaotanyan St., Shapingba District, Chongqing, China; [2]Medical Research Center, Southwest hospital, Third Military Medical University, 29# Gaotanyan St., Shapingba District, Chongqing, China.

Contents

Introduction....2
Benchmark information....2
  Softwares....2
  Datasets....2
  Platform....3
  Automatic benchmark and plotting scripts....3
Benchmark of computing complement base....4
Benchmark of FASTA/Q file parsing....5
  Datasets....5
  Tests....5
  Results....5
Performance of SeqKit with multiple threads....6
  Datasets....6
  Tests....6
  Results....7
Performance comparison with other tools....8
  Tests....8
  Results....9
Performance of SeqKit on different sizes of data....11
  Datasets....11
  Tests....11
  Results....11

Introduction

SeqKit is a cross-platform, ultrafast and practical command-line toolkit that is usable for researchers to complete wide range of FASTA/Q file processings. SeqKit is open source and available on Github at https://github.com/shenwei356/seqkit, with detailed usage, examples and tutorials at http://shenwei356.github.io/seqkit/

Benchmark information

Softwares

Four tools was used for performance comparison:

- seqkit. (Go). Version v0.3.1.1. Compiled with Go 1.7rc5.
- fasta_utilities. (Perl). Version 3dcc0bc.
- seqmagick. (Python). Version 0.6.1
- seqtk. (C). Version 1.1-r92-dirty.

A Python script memusg was used to compute running time and peak memory usage of a process.

Datasets

All test data is available here: seqkit-benchmark-data.tar.gz (2.2G)

1. **dataset_A.fa - large number of short sequences**

   Dataset A contains reference genomes DNA sequences of gastrointestinal tract from NIH Human Microbiome Project: Gastrointestinal_tract.nuc.fsa (FASTA format, ~2.7G). Rename it to dataset_A.fa.

2. **dataset_B.fa - small number of large sequences**

   Dataset B is Human genome from ensembl.

   - Genome DNA: Homo_sapiens.GRCh38.dna_sm.primary_assembly.fa.gz (Gzipped FASTA file, ~900M). Decompress it and rename to dataset_B.fa (~2.9G).
   - GTF file: Homo_sapiens.GRCh38.84.gtf.gz (~44M)
   - BED file: Homo_sapiens.GRCh38.84.bed.gz was converted from Homo_sapiens.GRCh38.84.gtf.gz by gtf2bed with command:

   ```
   $ zcat Homo_sapiens.GRCh38.84.gtf.gz | gtf2bed --do-not-sort | gzip -c >
   Homo_sapiens.GRCh38.84.bed.gz
   ```

3. **dataset_C.fq – Illumina single end reads (SE100)**

Dataset C is Illumina single end (SE 100bp) reads file (~2.2G).

Summary:

```
$ seqkit stat *.fa
file           format  type   num_seqs         sum_len  min_len        avg_len      max_len
dataset_A.fa   FASTA   DNA      67,748   2,807,643,808       56       41,442.5    5,976,145
dataset_B.fa   FASTA   DNA         194   3,099,750,718      970   15,978,096.5  248,956,422
dataset_C.fq   FASTQ   DNA   9,186,045     918,604,500      100            100          100
```

4. Sequence ID list

Parts of sequences IDs was sampled and shuffled from original data. They were used in test of extracting sequences by ID list.

```
$ seqkit sample -p 0.3 dataset_A.fa | seqkit seq --name --only-id | shuf > ids_A.txt
$ seqkit sample -p 0.3 dataset_B.fa | seqkit seq --name --only-id | shuf > ids_B.txt
$ seqkit sample -p 0.03 dataset_C.fq | seqkit seq --name --only-id | shuf > ids_C.txt
```

Summary:

```
$ wc -l ids*.txt
   20138 ids_A.txt
      58 ids_B.txt
  275467 ids_C.txt
```

5. BED file

Only BED data of chromosome 19 was used in test of subsequence with BED file:

```
$ zcat Homo_sapiens.GRCh38.84.bed.gz | grep -E "^19" | gzip -c > chr19.bed.gz
```

Platform

PC:

- CPU: Intel Core i5-3320M @ 2.60GHz, two cores/4 threads
- RAM: DDR3 1600MHz, 12GB
- SSD: 250G, SATA-3
- OS: Fedora 24 (Scientific KDE spin), Kernal: 4.6.4-301.fc24.x86_64

Software:

- Perl: perl 5, version 22, subversion 2 (v5.22.2) built for x86_64-linux-thread-multi
- Python: Python 2.7.11 (default, Jul 10 2016, 20:58:20) [GCC 6.1.1 20160621 (Red Hat 6.1.1-3)] on linux2

Automatic benchmark and plotting scripts

Scripts are available at https://github.com/shenwei356/seqkit/tree/master/benchmark .

Benchmark of computing complement base

To evaluate the performance of the algorithm for complementary base, a test was performed. Go source code is hosted at Github Gist and also in S2_File.zip.

Table A. Benchmark result of computing complement base.

Tests	Iterations	Time/operation
BenchmarkCheckLetterWithMap-4	2000000000	0.20 ns/op
BenchmarkCheckLetterWithSwitch-4	1000000000	0.03 ns/op
BenchmarkCheckLetterWithSwitchWithLargerAlphabetSize-4	2000000000	0.02 ns/op
**BenchmarkCheckLetterWithSlice-4**	2000000000	**0.01 ns/op**

Benchmark of FASTA/Q file parsing

Datasets

Previously described dataset A, B and C.

Tests

Source code is hosted at [Github](#) and also in S2_File.zip. Tests were repeated 5 times and average time and peak memory were used for plotting. All files were readed once before tests beginning to minimize the influence of page cache.

Note that seqtk does not support wrapped (fixed line width) output, so SeqKit uses "-w 0" to disable output wrapping.

Computation results were checked by file MD5 digest to ensure accuracy, which are available in corresponding result files.

Results

See figure 1 in manuscript.

Performance of SeqKit with multiple threads

Datasets

Same as previous datasets.

Tests

Similar to previous tests. ***Source code is hosted at [Github](#) and also in S2_File.zip***.

1. **Reverse Complement**
   Commands
2. **Extracting sequences by ID list**
   Commands
3. **Sampling by number**
   Commands
4. **Removing duplicates by sequence content**
   Commands
5. **Subsequence with BED file**
   Commands

Results

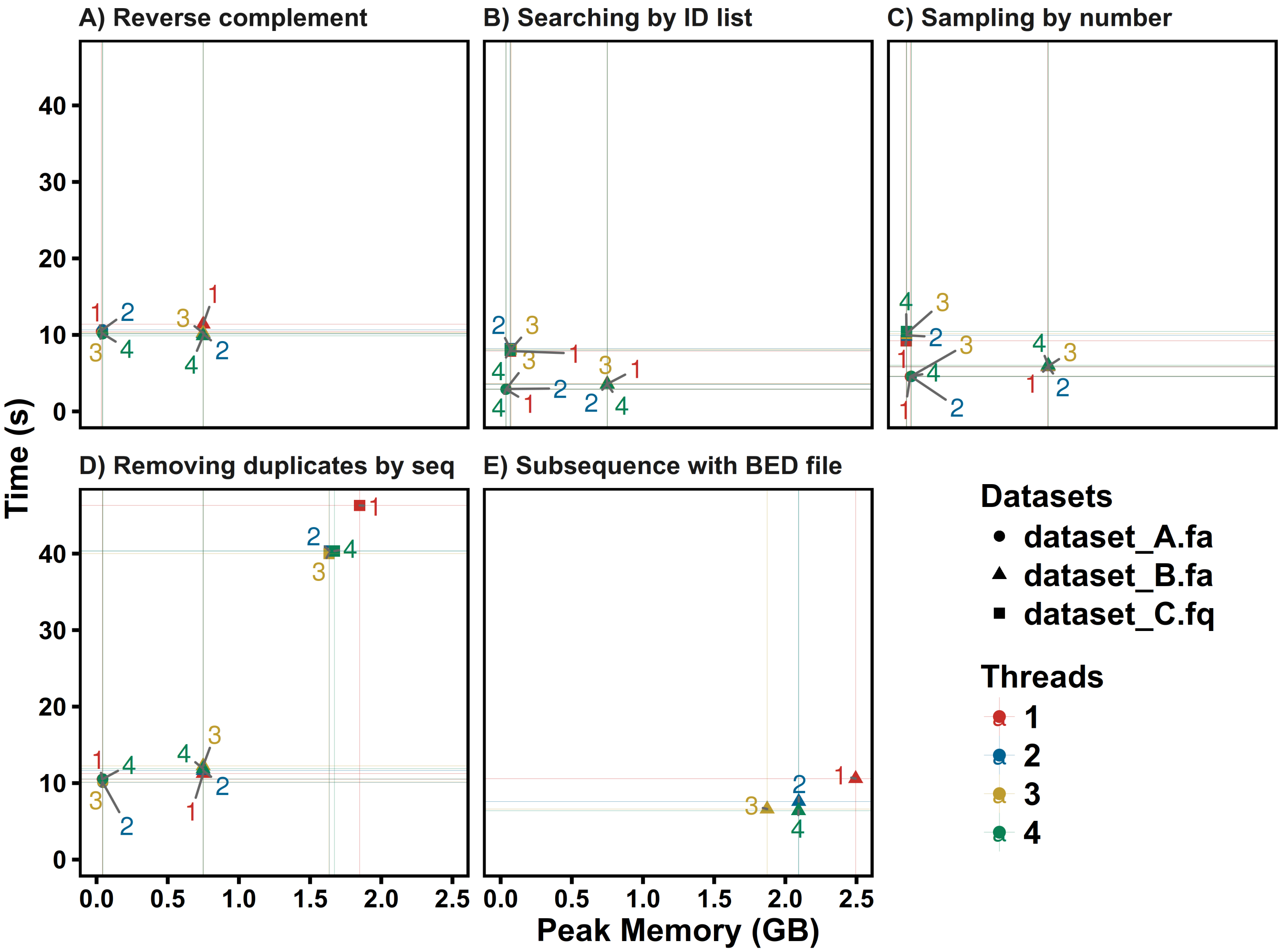

Figure A. Performances of SeqKit with multiple threads in five manipulations of FASTA/Q files. All tests were repeated three times and average time and peak memory were used for plotting.

Performance comparison with other tools

Tests

To evaluate the performance of SeqKit, several tests of common manipulations on FASTA/Q were performed. All tests was repeated 3 times and average time and peak memory were used for plotting. All files were readed once before tests beginning to minimize the influence of page cache.

Note that output sequences of all softwares were not wrapped to fixed length.

Computation results were checked by file MD5 digest and sequences statistics to ensure accuracy, which are available in corresponding result files.

Source code is hosted at Github and also in S2_File.zip.

1. **Reverse Complement**
   revcom_biogo (source, binary, compiled with Go 1.7rc5), a tool also written in Go using package biogo (Version 7ebd71b) is also used for comparison of FASTA file parsing performance.
   Note that some softwares (fasta_utilities and biogo) have different converting rules of computing complement sequence on ambiguous bases, therefore the results are different from others.
   Commands
2. **Extracting sequences by ID list**
   Commands
3. **Sampling by number**
   Note that different softwares have different sampling strategies, the peak memory depends on size of sampled sequences and the results may not be the same.
   Commands
4. **Removing duplicates by sequence content**
   Commands
5. **Subsequence with BED file**
   Commands

Results

Table B. Benchmark result of five manipulations

test	dataset	app	time_mean	time_stdev	mem_mean	mem_stdev
A) Reverse complement	dataset_A.fa	biogo	107.73	1.35	31496	841
A) Reverse complement	dataset_A.fa	fasta_utilities	16.91	0.28	52022	544
A) Reverse complement	dataset_A.fa	seqkit	10.93	0.58	44082	122
A) Reverse complement	dataset_A.fa	seqmagick	57.15	0.23	50841	1385
A) Reverse complement	dataset_A.fa	seqtk	8.90	0.12	7533	37
A) Reverse complement	dataset_B.fa	biogo	94.75	0.36	1325084	68795
A) Reverse complement	dataset_B.fa	fasta_utilities	24.02	0.37	1255725	62
A) Reverse complement	dataset_B.fa	seqkit	9.90	0.37	785510	115
A) Reverse complement	dataset_B.fa	seqmagick	64.00	0.29	1384210	89700
A) Reverse complement	dataset_B.fa	seqtk	9.63	0.06	244844	23
B) Searching by ID list	dataset_A.fa	fasta_utilities	9.65	0.11	52265	494
B) Searching by ID list	dataset_A.fa	seqkit	2.88	0.01	39518	114
B) Searching by ID list	dataset_A.fa	seqmagick	38.57	0.57	38998	1332
B) Searching by ID list	dataset_A.fa	seqtk	10.73	0.06	9901	1
B) Searching by ID list	dataset_B.fa	fasta_utilities	12.19	0.13	1255814	7
B) Searching by ID list	dataset_B.fa	seqkit	3.53	0.06	785308	122
B) Searching by ID list	dataset_B.fa	seqmagick	42.77	0.29	977886	69
B) Searching by ID list	dataset_B.fa	seqtk	13.02	0.10	244820	67
B) Searching by ID list	dataset_C.fq	fasta_utilities	86.42	2.58	77085	87
B) Searching by ID list	dataset_C.fq	seqkit	8.26	0.02	70129	905
B) Searching by ID list	dataset_C.fq	seqmagick	237.81	6.33	52580	76
B) Searching by ID list	dataset_C.fq	seqtk	6.16	0.12	39950	19
C) Sampling by number	dataset_A.fa	seqkit	4.60	0.17	48717	81
C) Sampling by number	dataset_A.fa	seqmagick	38.57	1.88	543829	8690
C) Sampling by number	dataset_A.fa	seqtk	4.13	0.04	1078777	1425
C) Sampling by number	dataset_B.fa	seqkit	5.90	0.19	1056069	73
C) Sampling by number	dataset_B.fa	seqmagick	42.59	1.00	2987453	31663
C) Sampling by number	dataset_B.fa	seqtk	5.97	0.34	2817729	33
C) Sampling by number	dataset_C.fq	seqkit	9.69	0.11	13405	912
C) Sampling by number	dataset_C.fq	seqmagick	284.56	4.89	3740949	36
C) Sampling by number	dataset_C.fq	seqtk	4.98	0.16	501608	42
D) Removing duplicates by seq	dataset_A.fa	seqkit	10.19	0.24	45918	128
D) Removing duplicates by seq	dataset_A.fa	seqmagick	49.98	0.48	54606	472
D) Removing duplicates by seq	dataset_B.fa	seqkit	11.12	0.29	785329	166
D) Removing duplicates by seq	dataset_B.fa	seqmagick	62.16	1.22	1033625	83
D) Removing duplicates by seq	dataset_C.fq	seqkit	40.22	0.18	1656700	152410
D) Removing duplicates by seq	dataset_C.fq	seqmagick	469.96	12.62	2737658	60
E) Subsequence with BED file	dataset_B.fa	seqkit	7.27	0.17	2218930	97457
E) Subsequence with BED file	dataset_B.fa	seqtk	6.42	0.11	246216	53

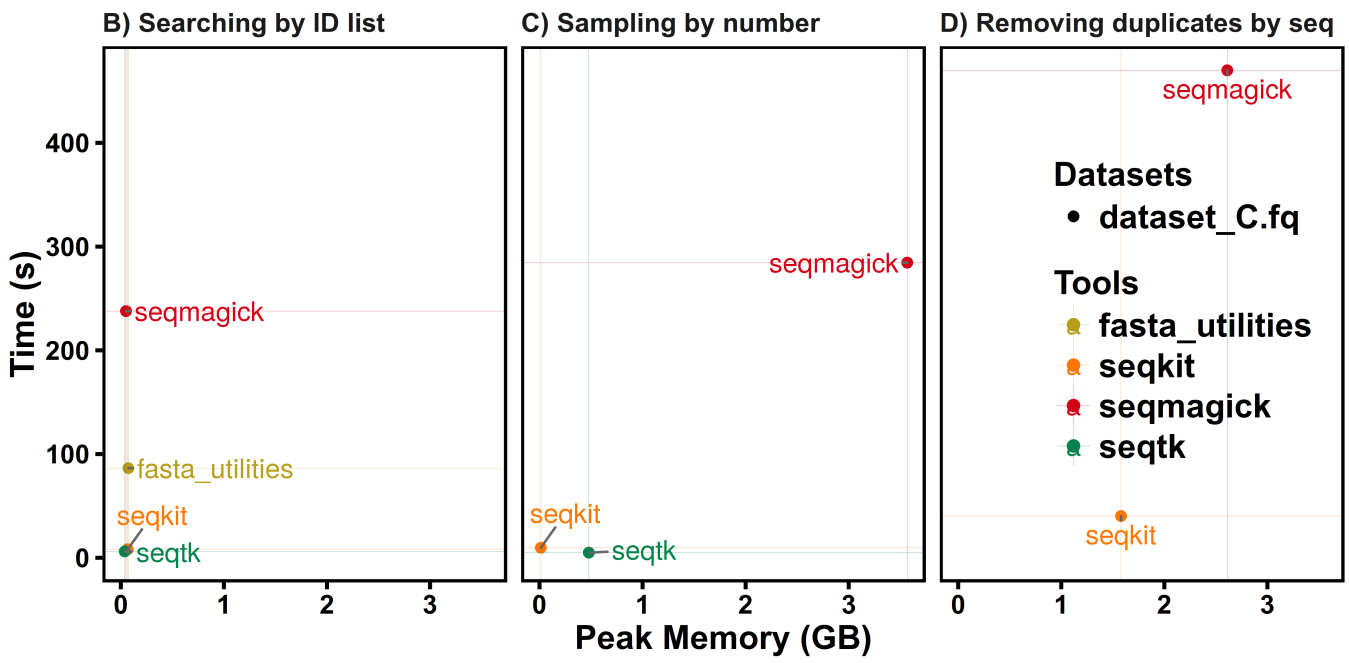

Figure B. Performance comparison on three manipulations of FASTQ file. All tests were repeated three times and average time and peak memory were used for plotting.

See Figure 2 in manuscript for performance comparison on three manipulations of FASTA files.

Performance of SeqKit on different sizes of data

Datasets

Sequences of Human genome chromosome 1 was extracted from dataset_B.fa as data of 1X (length: 248,956,422 bp, file size: 241.4Mb)

```
$ seqkit head -n 1 dataset_B.fa > 1X.fa
```

And it's replicated by *N* times as data of *N*X:

```
$ cat 1X.fa 1X.fa > 2X.fa
$ cat 2X.fa 2X.fa > 4X.fa
…
```

At last, rename the sequence identifiers so they could be rightly indexed.

```
for f in *X.fa; do seqkit rename $f > $f.re; mv $f.re $f; done
```

Tests

Four tests were performed. ***Source code is hosted at Github and also in S2_File.zip***.

1. **Reverse Complement**

   Commands

2. **Removing duplicates by sequence content**

   Commands

3. **Shuffling**

   Commands

4. **Sorting by length**

   Commands

Results

See Figure 3 in manuscript.
