# Supplementary material for: SeqKit: A Cross-Platform and Ultrafast Toolkit for FASTA/Q File Manipulation
Source: PLoS One. 2016 Oct 5;11(10):e0163962. doi: 10.1371/journal.pone.0163962 (PMC5051824; doi:10.1371/journal.pone.0163962)
Supplement: S2 File — All data supporting this article including source code, documents, executable binary files, benchmark scripts and plotting scripts. (ZIP) [file pone.0163962.s002.zip › SeqKit-supplementary-data2/doc/site/base.html]

{% if page\_description %}{% endif %}
{% if site\_author %}{% endif %}
{% if canonical\_url %}{% endif %}
{% if favicon %}
{% else %}{% endif %}
{% if page\_title %}{{ page\_title }} - {% endif %}{{ site\_name }}


{%- for path in extra\_css %}
{%- endfor %}

{% if google\_analytics %}
{% endif %}

{% include "nav.html" %}

{% block content %}

{% include "toc.html" %}

{% include "content.html" %}

{% endblock %}

---

{% if copyright %}

{{ copyright }}

{% endif %}

Documentation built with MkDocs.

{%- for path in extra\_javascript %}
{%- endfor %}

×Close

#### Search

From here you can search these documents. Enter
your search terms below.

{% if current\_page and current\_page.is\_homepage %}
{% endif %}
