# Supplementary material for: SeqKit: A Cross-Platform and Ultrafast Toolkit for FASTA/Q File Manipulation
Source: PLoS One. 2016 Oct 5;11(10):e0163962. doi: 10.1371/journal.pone.0163962 (PMC5051824; doi:10.1371/journal.pone.0163962)
Supplement: S2 File — All data supporting this article including source code, documents, executable binary files, benchmark scripts and plotting scripts. (ZIP) [file pone.0163962.s002.zip › SeqKit-supplementary-data2/doc/site/benchmark/index.html]

Benchmark - SeqKit - Ultrafast FASTA/Q kit


Toggle navigation


SeqKit - Ultrafast FASTA/Q kit

- Home
- Download
- Usage
- Tutorial
- Benchmark
- YanLi Lab

- Search
- Previous
- Next
- GitHub

- Softwares
- Features
- Datasets
- dataset\_A.fa - large number of short sequences
- dataset\_B.fa - small number of large sequences
- dataset\_C.fq – Illumina single end reads (SE100)
- Sequence ID list
- BED file
- Platform
- Tests
- Test 1. Reverse Complement
- Test 2. Extracting sequences by ID list
- Test 3. Sampling by number
- Test 4. Removing duplicates by sequence content
- Test 5. Subsequence with BED file
- Results
- Test of multiple threads:
- Tests on different file sizes

```
# Benchmark
```

## Softwares

1. seqkit. (Go).
   Version v0.3.1.1.
   Compiled with Go 1.7rc5.
2. fasta\_utilities. (Perl).
   Version 3dcc0bc.
   Lots of dependencies to install.
3. fastx\_toolkit. (Perl).
   Version 0.0.13.
   Can't handle multi-line FASTA files.
4. seqmagick. (Python).
   Version 0.6.1
5. seqtk. (C).
   Version 1.1-r92-dirty.

Not used:

1. pyfaidx. (Python).
   Version 0.4.7.1. *Not used, because it exhausted my memory (10G) when computing reverse-complement on a 5GB fasta file of 250 bp.*

A Python script memusg was used
to compute running time and peak memory usage of a process.

## Features

| Categories | Features | seqkit | fasta\_utilities | fastx\_toolkit | pyfaidx | seqmagick | seqtk |
| --- | --- | --- | --- | --- | --- | --- | --- |
| **Formats support** | Multi-line FASTA | Yes | Yes | -- | Yes | Yes | Yes |
|  | FASTQ | Yes | Yes | Yes | -- | Yes | Yes |
|  | Multi-line FASTQ | Yes | Yes | -- | -- | Yes | Yes |
|  | Validating sequences | Yes | -- | Yes | Yes | -- | -- |
|  | Supporting RNA | Yes | Yes | -- | -- | Yes | Yes |
| **Functions** | Searching by motifs | Yes | Yes | -- | -- | Yes | -- |
|  | Sampling | Yes | -- | -- | -- | Yes | Yes |
|  | Extracting sub-sequence | Yes | Yes | -- | Yes | Yes | Yes |
|  | Removing duplicates | Yes | -- | -- | -- | Partly | -- |
|  | Splitting | Yes | Yes | -- | Partly | -- | -- |
|  | Splitting by seq | Yes | -- | Yes | Yes | -- | -- |
|  | Shuffling | Yes | -- | -- | -- | -- | -- |
|  | Sorting | Yes | Yes | -- | -- | Yes | -- |
|  | Locating motifs | Yes | -- | -- | -- | -- | -- |
|  | Common sequences | Yes | -- | -- | -- | -- | -- |
|  | Cleaning bases | Yes | Yes | Yes | Yes | -- | -- |
|  | Transcription | Yes | Yes | Yes | Yes | Yes | Yes |
|  | Translation | -- | Yes | Yes | Yes | Yes | -- |
|  | Filtering by size | Indirect | Yes | -- | Yes | Yes | -- |
|  | Renaming header | Yes | Yes | -- | -- | Yes | Yes |
| **Other features** | Cross-platform | Yes | Partly | Partly | Yes | Yes | Yes |
|  | Reading STDIN | Yes | Yes | Yes | -- | Yes | Yes |
|  | Reading gzipped file | Yes | Yes | -- | -- | Yes | Yes |
|  | Writing gzip file | Yes | -- | -- | -- | Yes | -- |

**Note 2**: See usage for detailed options of seqkit.

## Datasets

All test data is available here: seqkit-benchmark-data.tar.gz (1.7G)

### dataset\_A.fa - large number of short sequences

Dataset A is reference genomes DNA sequences of gastrointestinal tract from
NIH Human Microbiome Project:
`Gastrointestinal_tract.nuc.fsa` (FASTA format, ~2.7G).

### dataset\_B.fa - small number of large sequences

Dataset B is Human genome from ensembl.

- Genome DNA: `Homo_sapiens.GRCh38.dna_sm.primary_assembly.fa.gz` (Gzipped FASTA file, ~900M)
  . Decompress it and rename to dataset\_B.fa (~2.9G).
- GTF file: `Homo_sapiens.GRCh38.84.gtf.gz` (~44M)
- BED file: `Homo_sapiens.GRCh38.84.bed.gz` was converted from `Homo_sapiens.GRCh38.84.gtf.gz` by `gtf2bed` with command

  ```
  $ zcat Homo_sapiens.GRCh38.84.gtf.gz | gtf2bed --do-not-sort | gzip -c > Homo_sapiens.GRCh38.84.bed.gz
  ```

### dataset\_C.fq – Illumina single end reads (SE100)

Dataset C is Illumina single end (SE 100bp) reads file (~2.2G).

Summary

```
$ seqkit stat *.fa
file          format  type   num_seqs        sum_len  min_len       avg_len      max_len
dataset_A.fa  FASTA   DNA      67,748  2,807,643,808       56      41,442.5    5,976,145
dataset_B.fa  FASTA   DNA         194  3,099,750,718      970  15,978,096.5  248,956,422
dataset_C.fq  FASTQ   DNA   9,186,045    918,604,500      100           100          100
```

### Sequence ID list

Parts of sequences IDs was sampled and shuffled from original data.
They were used in test of extracting sequences by ID list.

Commands:

```
$ seqkit sample -p 0.3  dataset_A.fa | seqkit seq --name --only-id | shuf > ids_A.txt
$ seqkit sample -p 0.3  dataset_B.fa | seqkit seq --name --only-id | shuf > ids_B.txt    
$ seqkit sample -p 0.03 dataset_C.fq | seqkit seq --name --only-id | shuf > ids_C.txt
```

Numbers:

```
$ wc -l ids*.txt
    20138 ids_A.txt
    58 ids_B.txt
2754516 ids_C.txt
```

### BED file

Only BED data of chromosome 19 was used in test of subsequence with BED file:

```
$ zcat Homo_sapiens.GRCh38.84.bed.gz | grep -E "^19" | gzip -c > chr19.bed.gz
```

## Platform

PC:

- CPU: Intel Core i5-3320M @ 2.60GHz, two cores/4 threads
- RAM: DDR3 1600MHz, 12GB
- SSD: SAMSUNG 850 EVO 250G, SATA-3
- OS: Fedora 24 (Scientific KDE spin), Kernal: 4.6.4-301.fc24.x86\_64

Softwares:

- Perl: perl 5, version 22, subversion 2 (v5.22.2) built for x86\_64-linux-thread-multi
- Python: Python 2.7.11 (default, Jul 10 2016, 20:58:20) [GCC 6.1.1 20160621 (Red Hat 6.1.1-3)] on linux2

## Tests

Automatic benchmark and plotting scripts are available at: https://github.com/shenwei356/seqkit/tree/master/benchmark.

All tests were repeated 3 times ( ~20 min for one time),
and average time and peak memory ware used for plotting.

All data were readed once before tests began to minimize the influence of page cache.

Output sequences of all softwares were not wrapped to fixed length.

### Test 1. Reverse Complement

`revcom_biogo` (source,
binary ),
a tool written in Golang (compiled with Go 1.6.3) using biogo
(Version 7ebd71b)
package,
was also used for comparison of FASTA file parsing performance.

*Note that some softwares (fasta\_utilities and biogo) have different converting rules of computing complement sequence on ambiguous bases, there fore the results are different from others.*

Commands

### Test 2. Extracting sequences by ID list

Commands

### Test 3. Sampling by number

*Note that different softwares have different sampling strategies, the peak memory depends on size of sampled sequences and the results may not be the same.*

Commands

### Test 4. Removing duplicates by sequence content

Commands

### Test 5. Subsequence with BED file

Commands

## Results

seqkit version: v0.3.1.1

FASTA:

FASTQ:

### Test of multiple threads:

From the results, 2 threads/CPU is enough, so the default threads of seqkit is 2.

### Tests on different file sizes

Files are generated by replicating Human genome chr1 for N times.

Please enable JavaScript to view the comments powered by Disqus.

---

Documentation built with MkDocs.

×Close

#### Search

From here you can search these documents. Enter
your search terms below.
