# Supplementary material for: SeqKit: A Cross-Platform and Ultrafast Toolkit for FASTA/Q File Manipulation
Source: PLoS One. 2016 Oct 5;11(10):e0163962. doi: 10.1371/journal.pone.0163962 (PMC5051824; doi:10.1371/journal.pone.0163962)
Supplement: S2 File — All data supporting this article including source code, documents, executable binary files, benchmark scripts and plotting scripts. (ZIP) [file pone.0163962.s002.zip › SeqKit-supplementary-data2/doc/site/download/index.html]

Download - SeqKit - Ultrafast FASTA/Q kit


Toggle navigation


SeqKit - Ultrafast FASTA/Q kit

- Home
- Download
- Usage
- Tutorial
- Benchmark
- YanLi Lab

- Search
- Previous
- Next
- GitHub

- Download
- Latest Version
- Installation
- Release History

# Download

SeqKit is implemented in Golang programming language,
executable binary files **for most popular operating systems** are freely available
in release page.

## Latest Version

SeqKit v0.3.1.1

***64-bit versions are highly recommended.***

### Links

- **Linux**
  - seqkit\_linux\_386.tar.gz
  - seqkit\_linux\_amd64.tar.gz
  - seqkit\_linux\_arm.tar.gz
- **Mac OS X**
  - seqkit\_darwin\_386.tar.gz
  - seqkit\_darwin\_amd64.tar.gz
- **Windows**
  - seqkit\_windows\_386.exe.tar.gz
  - seqkit\_windows\_amd64.exe.tar.gz
- **FreeBSD**
  - seqkit\_freebsd\_386.tar.gz
  - seqkit\_freebsd\_amd64.tar.gz
  - seqkit\_freebsd\_arm.tar.gz
- **OpenBSD**
  - seqkit\_openbsd\_386.tar.gz
  - seqkit\_openbsd\_amd64.tar.gz

### Mirror site for Chinese user

- **Linux**
  - seqkit\_linux\_386.tar.gz
  - seqkit\_linux\_amd64.tar.gz
  - seqkit\_linux\_arm.tar.gz
- **Mac OS X**
  - seqkit\_darwin\_386.tar.gz
  - seqkit\_darwin\_amd64.tar.gz
- **Windows**
  - seqkit\_windows\_386.exe.tar.gz
  - seqkit\_windows\_amd64.exe.tar.gz
- **FreeBSD**
  - seqkit\_freebsd\_386.tar.gz
  - seqkit\_freebsd\_amd64.tar.gz
  - seqkit\_freebsd\_arm.tar.gz
- **OpenBSD**
  - seqkit\_openbsd\_386.tar.gz
  - seqkit\_openbsd\_amd64.tar.gz

## Installation

Just download compressed
executable file of your operating system,
and uncompress it with `tar -zxvf *.tar.gz` command or other tools.
And then:

1. **For Linux-like systems**

   1. If you have root privilege simply copy it to `/usr/local/bin`:

      ```
      sudo cp seqkit /usr/local/bin/
      ```
   2. Or add the directory of the executable file to environment variable
      `PATH`:

      ```
      echo export PATH=\$PATH:/PATH/OF/seqkit >> ~/.bashrc
      ```
2. **For windows**, just copy `seqkit.exe` to `C:\WINDOWS\system32`.

For Go developer, just one command:

```
go get -u github.com/shenwei356/seqkit/seqkit
```

## Release History

- SeqKit v0.3.1.1
  - compile with go1.7rc5, with ***higher performance and smaller size of binary file***
- SeqKit v0.3.1
  - improve speed of `seqkit locate`
- SeqKit v0.3.0
  - use fork of github.com/brentp/xopen, using `zcat` for speedup of .gz file
    reading on \*nix systems.
  - improve speed of parsing sequence ID when creating FASTA index
  - reduce memory usage of `seqkit subseq --gtf`
  - fix bug of `seqkit subseq` when using flag `--id-ncbi`
  - fix bug of `seqkit split`, outdir error
  - fix bug of `seqkit seq -p`, last base is wrongly failed to convert when
    sequence length is odd.
  - add "sum\_len" result for output of `seqkit stat`
- seqkit v0.2.9
  - fix minor bug of `seqkit split` and `seqkit shuffle`,
    header name error due to improper use of pointer
  - add option `-O (--out-dir)` to `seqkit split`
- seqkit v0.2.8
  - improve speed of parsing sequence ID, not using regular expression for default `--id-regexp`
  - improve speed of record outputing for small-size sequences
  - fix minor bug: `seqkit seq` for blank record
  - update benchmark result
- seqkit v0.2.7
  - ***reduce memory usage*** by optimize the outputing of sequences.
    detail: using `BufferedByteSliceWrapper` to resuse bytes.Buffer.
  - ***reduce memory usage and improve speed*** by using custom buffered
    reading mechanism, instead of using standard library `bufio`,
    which is slow for large genome sequence.
  - discard strategy of "buffer" and "chunk" of FASTA/Q records,
    just parse records one by one.
  - delete global flags `-c (--chunk-size)` and `-b (--buffer-size)`.
  - add function testing scripts
- seqkit v0.2.6
  - fix bug of `seqkit subseq`: Inplace subseq method leaded to wrong result
- seqkit v0.2.5.1
  - fix a bug of `seqkit subseq`: chromesome name was not be converting to lower case when using `--gtf` or `--bed`
- seqkit v0.2.5
  - fix a serious bug brought in `v0.2.3`, using unsafe method to convert `string` to `[]byte`
  - add awk-like built-in variable of record number (`{NR}`) for `seqkit replace`
- seqkit v0.2.4.1
  - fix several bugs from library `bio`, affected situations:
    - Locating patterns in sequences by pattern FASTA file: `seqkit locate -f`
    - Reading FASTQ file with record of which the quality starts with `+`
  - add command `version`
- seqkit v0.2.4
  - add subcommand `head`
- seqkit v0.2.3
  - reduce memory occupation by avoid copy data when convert `string` to `[]byte`
  - speedup reverse-complement by avoid repeatly calling functions
- seqkit v0.2.2
  - reduce memory occupation of subcommands that use FASTA index
- seqkit v0.2.1
  - improve performance of outputing.
  - fix bug of `seqkit seq -g` for FASTA fromat
  - some other minor fix of code and docs
  - update benchmark results
- seqkit v0.2.0
  - ***reduce memory usage of writing output***
  - fix bug of `subseq`, `shuffle`, `sort` when reading from stdin
  - reduce memory usage of `faidx`
  - make validating sequences an optional option in `seq` command, it saves some time.
- seqkit v0.1.9
  - using custom FASTA index file extension: `.seqkit.fai`
  - reducing memory usage of `sample --number --two-pass`
  - ***change default CPU number to 2 for multi-cpus computer, and 1 for single-CPU computer***
- seqkit v0.1.8
  - add subcommand `rename` to rename duplicated IDs
  - add subcommand `faidx` to create FASTA index file
  - ***utilize faidx to improve performance of `subseq`***
  - *`shuffle`, `sort` and split support two-pass mode (by flag `-2`) with faidx to reduce memory usage.*
  - document update
- seqkit v0.1.7
  - ***add support for (multi-line) FASTQ format***
  - update document, add technical details
  - rename subcommands `fa2tab` and `tab2fa` to `fx2tab` and `tab2fx`
  - add subcommand `fq2fa`
  - add column "seq\_format" to `stat`
  - add global flag `-b` (`--bufer-size`)
  - little change of flag in `subseq` and some other commands
- seqkit v0.1.6
  - add subcommand `replace`
- seqkit v0.1.5.2
  - fix bug of `grep`, when not using flag `-r`, flag `-i` will not take effect.
- seqkit v0.1.5.1
  - fix result of `seqkit sample -n`
  - fix benchmark script
- seqkit v0.1.5
  - add global flag `--id-ncbi`
  - add flag `-d` (`--dup-seqs-file`) and `-D` (`--dup-num-file`) for subcommand `rmdup`
  - make using MD5 as an optional flag `-m` (`--md5`) in subcommand `rmdup` and `common`
  - fix file name suffix of `seqkit split` result
  - minor modification of `sliding` output
- seqkit v0.1.4.1
  - change alignment of `stat` output
  - preciser CPUs number control
- seqkit v0.1.4
  - add subcommand `sort`
  - improve subcommand `subseq`: supporting of getting subsequences by GTF and BED files
  - change name format of `sliding` result
  - prettier output of `stat`
- seqkit v0.1.3.1
  - Performance improvement by reducing time of cleaning spaces
  - Document update
- seqkit v0.1.3
  - **Further performance improvement**
  - Rename sub command `extract` to `grep`
  - Change default value of flag `--threads` back CPU number of current device,
    change default value of flag `--chunk-size` back 10000 sequences.
  - Update benchmark
- seqkit v0.1.2
  - Add flag `--dna2rna` and `--rna2dna` to subcommand `seq`.
- seqkit v0.1.1
  - **5.5X speedup of FASTA file parsing** by avoid using regular expression to remove spaces (detail ) and using slice indexing instead of map to validate letters (detail)
  - Change default value of global flag `-- thread` to 1. Since most of the subcommands are I/O intensive, For computation intensive jobs, like extract and locate, you may set a bigger value.
  - Change default value of global flag `--chunk-size` to 100.
  - Add subcommand `stat`
  - Fix bug of failing to automatically detect alphabet when only one record in file.
- seqkit v0.1
  - first release of seqkit

Please enable JavaScript to view the comments powered by Disqus.

---

Documentation built with MkDocs.

×Close

#### Search

From here you can search these documents. Enter
your search terms below.
