# Supplementary material for: SeqKit: A Cross-Platform and Ultrafast Toolkit for FASTA/Q File Manipulation
Source: PLoS One. 2016 Oct 5;11(10):e0163962. doi: 10.1371/journal.pone.0163962 (PMC5051824; doi:10.1371/journal.pone.0163962)
Supplement: S2 File — All data supporting this article including source code, documents, executable binary files, benchmark scripts and plotting scripts. (ZIP) [file pone.0163962.s002.zip › SeqKit-supplementary-data2/doc/site/example/index.html]

Example - seqkit - FASTA kit


Toggle navigation


seqkit - FASTA kit

- Home
- Download
- Manual
- Example
- About

- Search
- Previous
- Next
- GitHub

---

Documentation built with MkDocs.

×Close

#### Search

From here you can search these documents. Enter
your search terms below.
