# Supplementary material for: SeqKit: A Cross-Platform and Ultrafast Toolkit for FASTA/Q File Manipulation
Source: PLoS One. 2016 Oct 5;11(10):e0163962. doi: 10.1371/journal.pone.0163962 (PMC5051824; doi:10.1371/journal.pone.0163962)
Supplement: S2 File — All data supporting this article including source code, documents, executable binary files, benchmark scripts and plotting scripts. (ZIP) [file pone.0163962.s002.zip › SeqKit-supplementary-data2/doc/site/index.html]

SeqKit - Ultrafast FASTA/Q kit


Toggle navigation


SeqKit - Ultrafast FASTA/Q kit

- Home
- Download
- Usage
- Tutorial
- Benchmark
- YanLi Lab

- Search
- Previous
- Next
- GitHub

- SeqKit - a cross-platform and ultrafast toolkit for FASTA/Q file manipulation
- Introduction
- Features
- Installation
- Subcommands
- Technical details and guides for use
- Usage && Examples
- Benchmark
- Citation
- Contact
- License

# SeqKit - a cross-platform and ultrafast toolkit for FASTA/Q file manipulation

Documents: http://shenwei356.github.io/seqkit

Source code: https://github.com/shenwei356/seqkit

Latest version:

## Introduction

FASTA and FASTQ are basic formats for storing nucleotide and protein sequences.
The manipulations of FASTA/Q file include converting, clipping, searching,
filtering, deduplication, splitting, shuffling, sampling and so on.
Existed tools only implemented parts of the functions,
and some of them are only available for specific operating systems.
Furthermore, the complicated installation process of dependencies packages and
running environment also make them less friendly to common users.

SeqKit is a cross-platform, ultrafast, and practical FASTA/Q manipulations tool
that is friendly for researchers to complete wide ranges of FASTA/Q file processing.
The toolkit supports plain or gzip-compressed input and output
from either standard stream or files,
therefore, it could be easily used in command-line pipe.

## Features

- **Cross-platform** (Linux/Windows/Mac OS X/OpenBSD/FreeBSD,
  see download)
- **Light weight and out-of-the-box, no dependencies, no compilation, no configuration**
  (see download)
- **Very Fast** (see benchmark),
  **multiple-CPUs supported**.
- **Practical functions supported by 20 subcommands** (see subcommands and
  usage )
- **Well documented** (detailed usage
  and benchmark )
- **Seamlessly parses both FASTA and FASTQ formats**
- **Support STDIN and gziped input/output file, easy being used in pipe**
- **Support custom sequence ID regular expression** (especially useful for searching with ID list)
- Reproducible results (configurable rand seed in `sample` and `shuffle`)
- Well organized source code, friendly to use and easy to extend.

### Features comparison

| Categories | Features | seqkit | fasta\_utilities | fastx\_toolkit | pyfaidx | seqmagick | seqtk |
| --- | --- | --- | --- | --- | --- | --- | --- |
| **Formats support** | Multi-line FASTA | Yes | Yes | -- | Yes | Yes | Yes |
|  | FASTQ | Yes | Yes | Yes | -- | Yes | Yes |
|  | Multi-line FASTQ | Yes | Yes | -- | -- | Yes | Yes |
|  | Validating sequences | Yes | -- | Yes | Yes | -- | -- |
|  | Supporting RNA | Yes | Yes | -- | -- | Yes | Yes |
| **Functions** | Searching by motifs | Yes | Yes | -- | -- | Yes | -- |
|  | Sampling | Yes | -- | -- | -- | Yes | Yes |
|  | Extracting sub-sequence | Yes | Yes | -- | Yes | Yes | Yes |
|  | Removing duplicates | Yes | -- | -- | -- | Partly | -- |
|  | Splitting | Yes | Yes | -- | Partly | -- | -- |
|  | Splitting by seq | Yes | -- | Yes | Yes | -- | -- |
|  | Shuffling | Yes | -- | -- | -- | -- | -- |
|  | Sorting | Yes | Yes | -- | -- | Yes | -- |
|  | Locating motifs | Yes | -- | -- | -- | -- | -- |
|  | Common sequences | Yes | -- | -- | -- | -- | -- |
|  | Cleaning bases | Yes | Yes | Yes | Yes | -- | -- |
|  | Transcription | Yes | Yes | Yes | Yes | Yes | Yes |
|  | Translation | -- | Yes | Yes | Yes | Yes | -- |
|  | Filtering by size | Indirect | Yes | -- | Yes | Yes | -- |
|  | Renaming header | Yes | Yes | -- | -- | Yes | Yes |
| **Other features** | Cross-platform | Yes | Partly | Partly | Yes | Yes | Yes |
|  | Reading STDIN | Yes | Yes | Yes | -- | Yes | Yes |
|  | Reading gzipped file | Yes | Yes | -- | -- | Yes | Yes |
|  | Writing gzip file | Yes | -- | -- | -- | Yes | -- |

**Note 1**: See version information of the softwares.

**Note 2**: See usage for detailed options of seqkit.

## Installation

Go to Download Page for more download options and changelogs.

`seqkit` is implemented in Golang programming language,
executable binary files **for most popular operating systems** are freely available
in release page.

Just download compressed
executable file of your operating system,
and uncompress it with `tar -zxvf *.tar.gz` command or other tools.
And then:

1. **For Linux-like systems**

   1. If you have root privilege simply copy it to `/usr/local/bin`:

      ```
      sudo cp csvtk /usr/local/bin/
      ```
   2. Or add the directory of the executable file to environment variable
      `PATH`:

      ```
      echo export PATH=\$PATH:/PATH/OF/csvtk >> ~/.bashrc
      ```
2. **For windows**, just copy `csvtk.exe` to `C:\WINDOWS\system32`.

For Go developer, just one command:

```
go get -u github.com/shenwei356/csvtk/csvtk
```

## Subcommands

20 subcommands in total.

**Sequence and subsequence**

- `seq` transform sequences (revserse, complement, extract ID...)
- `subseq` get subsequences by region/gtf/bed, including flanking sequences
- `sliding` sliding sequences, circular genome supported
- `stat` simple statistics of FASTA files
- `faidx` create FASTA index file

**Format conversion**

- `fx2tab` covert FASTA/Q to tabular format (and length/GC content/GC skew)
- `tab2fx` covert tabular format to FASTA/Q format
- `fq2fa` covert FASTQ to FASTA

**Searching**

- `grep` search sequences by pattern(s) of name or sequence motifs
- `locate` locate subsequences/motifs

**Set operations**

- `rmdup` remove duplicated sequences by id/name/sequence
- `common` find common sequences of multiple files by id/name/sequence
- `split` split sequences into files by id/seq region/size/parts
- `sample` sample sequences by number or proportion
- `head` print first N FASTA/Q records

**Edit**

- `replace` replace name/sequence by regular expression
- `rename` rename duplicated IDs

**Ordering**

- `shuffle` shuffle sequences
- `sort` sort sequences by id/name/sequence

**Misc**

- `version` print version information and check for update

## Technical details and guides for use

### FASTA/Q format parsing

SeqKit uses author's lightweight and high-performance bioinformatics packages
bio for FASTA/Q parsing,
which has high performance
close to the
famous C lib klib (kseq.h).

### Sequence formats and types

SeqKit seamlessly support FASTA and FASTQ format.
Sequence format is automatically detected.
All subcommands except for `faidx` can handle both formats.
And only when some commands (`subseq`, `split`, `sort` and `shuffle`)
which utilise FASTA index to improve perfrmance for large files in two pass mode
(by flag `--two-pass`), only FASTA format is supported.

Sequence type (DNA/RNA/Protein) is automatically detected by leading subsequences
of the first sequences in file or STDIN. The length of the leading subsequences
is configurable by global flag `--alphabet-guess-seq-length` with default value
of 10000. If length of the sequences is less than that, whole sequences will
be checked.

### Sequence ID

By default, most softwares, including `seqkit`, take the leading non-space
letters as sequence identifier (ID). For example,

| FASTA header | ID |
| --- | --- |
| >123456 gene name | 123456 |
| >longname | longname |
| >gi|110645304|ref|NC\_002516.2| Pseudomona | gi|110645304|ref|NC\_002516.2| |

But for some sequences from NCBI,
e.g. `>gi|110645304|ref|NC_002516.2| Pseudomona`, the ID is `NC_002516.2`.
In this case, we could set sequence ID parsing regular expression by global flag
`--id-regexp "\|([^\|]+)\| "` or just use flag `--id-ncbi`. If you want
the `gi` number, then use `--id-regexp "^gi\|([^\|]+)\|"`.

### FASTA index

For some commands, including `subseq`, `split`, `sort` and `shuffle`,
when input files are (plain or gzipped) FASTA files,
FASTA index would be optional used for
rapid access of sequences and reducing memory occupation.

ATTENTION: the `.seqkit.fai` file created by SeqKit is a little different from `.fai` file
created by `samtools`. SeqKit uses full sequence head instead of just ID as key.

### Parallelization of CPU intensive jobs

The validation of sequences bases and complement process of sequences
are parallelized for large sequences.

Parsing of line-based files, including BED/GFF file and ID list file are also parallelized.

The Parallelization is implemented by multiple goroutines in golang
which are similar to but much
lighter weight than threads. The concurrency number is configurable with global
flag `-j` or `--threads` (default value: 1 for single-CPU PC, 2 for others).

### Memory occupation

Most of the subcommands do not read whole FASTA/Q records in to memory,
including `stat`, `fq2fa`, `fx2tab`, `tab2fx`, `grep`, `locate`, `replace`,
`seq`, `sliding`, `subseq`.

Note that when using `subseq --gtf | --bed`, if the GTF/BED files are too
big, the memory usage will increase.
You could use `--chr` to specify chromesomes and `--feature` to limit features.

Some subcommands need to store sequences or heads in memory, but there are
strategy to reduce memory occupation, including `rmdup` and `common`.
When comparing with sequences, MD5 digest could be used to replace sequence by
flag `-m` (`--md5`).

Some subcommands could either read all records or read the files twice by flag
`-2` (`--two-pass`), including `sample`, `split`, `shuffle` and `sort`.
They use FASTA index for rapid acccess of sequences and reducing memory occupation.

### Reproducibility

Subcommands `sample` and `shuffle` use random function, random seed could be
given by flag `-s` (`--rand-seed`). This makes sure that sampling result could be
reproduced in different environments with same random seed.

## Usage && Examples

Usage and examples

Tutorial

## Benchmark

More details: http://shenwei356.github.io/seqkit/benchmark/

Datasets:

```
$ seqkit stat *.fa
file          format  type   num_seqs        sum_len  min_len       avg_len      max_len
dataset_A.fa  FASTA   DNA      67,748  2,807,643,808       56      41,442.5    5,976,145
dataset_B.fa  FASTA   DNA         194  3,099,750,718      970  15,978,096.5  248,956,422
dataset_C.fq  FASTQ   DNA   9,186,045    918,604,500      100           100          100
```

SeqKit version: v0.3.1.1

FASTA:

FASTQ:

## Citation

**W Shen**, S Le, Y Li\*, F Hu\*. SeqKit: a cross-platform and ultrafast toolkit for FASTA/Q file manipulation.
***PLOS ONE***. revised.

## Contact

Email me for any problem when using seqkit. shenwei356(at)gmail.com

Create an issue to report bugs,
propose new functions or ask for help.

## License

MIT License

---

Documentation built with MkDocs.

×Close

#### Search

From here you can search these documents. Enter
your search terms below.
