# Supplementary material for: SeqKit: A Cross-Platform and Ultrafast Toolkit for FASTA/Q File Manipulation
Source: PLoS One. 2016 Oct 5;11(10):e0163962. doi: 10.1371/journal.pone.0163962 (PMC5051824; doi:10.1371/journal.pone.0163962)
Supplement: S2 File — All data supporting this article including source code, documents, executable binary files, benchmark scripts and plotting scripts. (ZIP) [file pone.0163962.s002.zip › SeqKit-supplementary-data2/doc/site/nav-sub.html]

{% if not nav\_item.children %}- {{ nav\_item.title }}
{% else %}- {{ nav\_item.title }}
  {% for nav\_item in nav\_item.children %}
  {% include "nav-sub.html" %}
  {% endfor %}
{% endif %}
