# Supplementary material for: SeqKit: A Cross-Platform and Ultrafast Toolkit for FASTA/Q File Manipulation
Source: PLoS One. 2016 Oct 5;11(10):e0163962. doi: 10.1371/journal.pone.0163962 (PMC5051824; doi:10.1371/journal.pone.0163962)
Supplement: S2 File — All data supporting this article including source code, documents, executable binary files, benchmark scripts and plotting scripts. (ZIP) [file pone.0163962.s002.zip › SeqKit-supplementary-data2/doc/site/nav.html]

{% if include\_nav or include\_next\_prev or repo\_url %}

Toggle navigation

{% endif %}
{{ site\_name }}

{% if include\_nav %}

{% for nav\_item in nav %}
{% if nav\_item.children %}- {{ nav\_item.title }} 
  {% for nav\_item in nav\_item.children %}
  {% include "nav-sub.html" %}
  {% endfor %}
{% else %}- {{ nav\_item.title }}
{% endif %}
{% endfor %}
{% endif %}

- Search
{% if include\_next\_prev %}- Previous
- Next
{% endif %}
{% if repo\_url %}- {% if repo\_name == 'GitHub' %}
  {% elif repo\_name == 'Bitbucket' %}
  {% endif %}
  {{ repo\_name }}
{% endif %}
