# Supplementary material for: SeqKit: A Cross-Platform and Ultrafast Toolkit for FASTA/Q File Manipulation
Source: PLoS One. 2016 Oct 5;11(10):e0163962. doi: 10.1371/journal.pone.0163962 (PMC5051824; doi:10.1371/journal.pone.0163962)
Supplement: S2 File — All data supporting this article including source code, documents, executable binary files, benchmark scripts and plotting scripts. (ZIP) [file pone.0163962.s002.zip › SeqKit-supplementary-data2/doc/site/tutorial/index.html]

Tutorial - SeqKit - Ultrafast FASTA/Q kit


Toggle navigation


SeqKit - Ultrafast FASTA/Q kit

- Home
- Download
- Usage
- Tutorial
- Benchmark
- YanLi Lab

- Search
- Previous
- Next
- GitHub

- Tutorial
- Some manipulations on big genomes
- Remove contaminated reads
- Handling of aligned sequences
- Play with miRNA hairpins
- Bacteria genome

# Tutorial

## Some manipulations on big genomes

A script memusg is
used to check the peek memory usage of seqkit. Usage: `memusg [-t] command`.

1. Human genome

   ```
   $ seqkit stat hsa.fa
   file    format  type  num_seqs        sum_len  min_len       avg_len      max_len
   hsa.fa  FASTA   DNA        194  3,099,750,718      970  15,978,096.5  248,956,422
   ```
2. Build FASTA index (***optional***, when using flag `-2` (`--two-pass`),
   some commands will automaticlly build it).
   For some commands, including `subseq`, `split`, `sort` and `shuffle`,
   when input files are (plain or gzipped) FASTA files or stdin,
   FASTA index would be optional used for
   rapid acccess of sequences and reducing memory occupation.
   ***ATTENTION***: the `.seqkit.fai` file created by SeqKit is a little different from .fai file
   created by samtools. SeqKit uses full sequence head instead of just ID as key.

   ```
   $ memusg -t seqkit faidx --id-regexp "^(.+)$"  hsa.fa -o hsa.fa.seqkit.fai

   elapsed time: 10.011s
   peak rss: 177.21 MB
   ```

   Create common .fai file:

   ```
   $ memusg -t seqkit faidx hsa.fa -o hsa.fa.fai2

   elapsed time: 10.454s
   peak rss: 172.82 MB
   ```

   Performance of samtools:

   ```
   $ memusg -t samtools faidx hsa.fa

   elapsed time: 9.574s
   peak rss: 1.45 MB
   ```

   Exactly same content:

   ```
   $ md5sum hsa.fa.fai*
   21e0c25b4d817d1c19ee8107191b9b31  hsa.fa.fai
   21e0c25b4d817d1c19ee8107191b9b31  hsa.fa.fai2
   ```
3. Sorting by sequence length

   ```
   $ memusg -t seqkit sort --by-length --reverse --two-pass hsa.fa > hsa.sorted.fa
   [INFO] create and read FASTA index ...
   [INFO] read sequence IDs and lengths from FASTA index ...
   [INFO] 194 sequences loaded
   [INFO] sorting ...
   [INFO] output ...

   elapsed time: 4.892s
   peak rss: 500.15 MB
   ```

   Detail:

   ```
   $ seqkit fx2tab --length hsa.sorted.fa --name --only-id | cut -f 1,4 | more
   1       248956422
   2       242193529
   3       198295559
   4       190214555
   5       181538259
   6       170805979
   7       159345973
   X       156040895
   8       145138636
   9       138394717
   11      135086622
   10      133797422
   12      133275309
   13      114364328
   14      107043718
   15      101991189
   16      90338345
   17      83257441
   18      80373285
   20      64444167
   19      58617616
   Y       57227415
   22      50818468
   21      46709983
   KI270728.1      1872759
   KI270727.1      448248
   ...

   real    0m10.697s
   user    0m11.153s
   sys     0m0.917s
   ```
4. Shuffling sequences

   ```
   $ memusg -t seqkit shuffle hsa.fa --two-pass > hsa.shuffled.fa
   [INFO] create and read FASTA index ...
   [INFO] read sequence IDs from FASTA index ...
   [INFO] 194 sequences loaded
   [INFO] shuffle ...
   [INFO] output ...

   elapsed time: 6.632s
   peak rss: 528.3 MB
   ```
5. Spliting into files with single sequence

   ```
   $ memusg -t seqkit split --by-id hsa.fa --two-pass
   [INFO] split by ID. idRegexp: ^([^\s]+)\s?
   [INFO] create and read FASTA index ...
   [INFO] read sequence IDs from FASTA index ...
   [INFO] 194 sequences loaded
   [INFO] write 1 sequences to file: hsa.id_KI270743.1.fa
   [INFO] write 1 sequences to file: hsa.id_KI270706.1.fa
   [INFO] write 1 sequences to file: hsa.id_KI270717.1.fa
   [INFO] write 1 sequences to file: hsa.id_KI270718.1.fa
   [INFO] write 1 sequences to file: hsa.id_KI270468.1.fa
   ...

   elapsed time: 18.807s
   peak rss: 1.36 GB
   ```
6. Geting subsequence of some chromesomes

   ```
   $ memusg -t seqkit subseq -r 1:10 --chr X --chr Y  hsa.fa
   >X_1-10 X dna_sm:chromosome chromosome:GRCh38:X:1:156040895:1 REF
   nnnnnnnnnn
   >Y_1-10 Y dna_sm:chromosome chromosome:GRCh38:Y:2781480:56887902:1 REF
   NNNNNNNNNN

   elapsed time: 1.276s
   peak rss: 640.92 MB
   ```
7. Geting CDS sequence of chr 1 by GTF files

   ```
   $ memusg -t seqkit subseq --gtf Homo_sapiens.GRCh38.84.gtf.gz --chr X --feature cds  hsa.fa > chrX.gtf.cds.fa
   [INFO] read GTF file ...
   [INFO] 22420 GTF features loaded

   elapsed time: 8.643s
   peak rss: 846.14 MB
   ```

## Remove contaminated reads

1. Mapping with reads on some potential contaminate genomes, and get the reads IDs list.

   ```
   $ wc -l contaminate.list
   244 contaminate.list

   $ head -n 2 contaminate.list
   HWI-D00523:240:HF3WGBCXX:1:1101:2574:2226
   HWI-D00523:240:HF3WGBCXX:1:1101:12616:2205
   ```
2. Remove contaminated reads

   ```
   $ seqkit grep -f contaminate.list -v reads_1.fq.gz -o reads_1.clean.fq.gz
   $ seqkit grep -f contaminate.list -v reads_2.fq.gz -o reads_2.clean.fq.gz

   $ seqkit stat *.fq.gz
   file                  seq_format   seq_type   num_seqs   min_len   avg_len   max_len
   reads_1.clean.fq.gz   FASTQ        DNA           2,256       226       227       229
   reads_1.fq.gz         FASTQ        DNA           2,500       226       227       229
   reads_2.clean.fq.gz   FASTQ        DNA           2,256       223       224       225
   reads_2.fq.gz         FASTQ        DNA           2,500       223       224       225
   ```

## Handling of aligned sequences

1. Some mock sequences (usually they will be much longer)

   ```
   $ cat seqs.fa
   >seq1
   ACAACGTCTACTTACGTTGCATCGTCATGCTGCATTACGTAGTCTGATGATG
   >seq2
   ACACCGTCTACTTTCATGCTGCATTACGTAGTCTGATGATG
   >seq3
   ACAACGTCTACTTACGTTGCATCGTCATGCTGCACTGATGATG
   >seq4
   ACAACGTCTACTTACGTTGCATCTTCGGTCATGCTGCATTACGTAGTCTGATGATG
   ```
2. Run multiple sequence alignment (clustalo)

   ```
   clustalo -i seqs.fa -o seqs.msa.fa --force --outfmt fasta --threads=4
   ```
3. Convert FASTA format to tabular format.

   ```
   $ seqkit fx2tab seqs.msa.fa
   seq1    ACAACGTCTACTTACGTTGCAT----CGTCATGCTGCATTACGTAGTCTGATGATG
   seq2    ---------------ACACCGTCTACTTTCATGCTGCATTACGTAGTCTGATGATG
   seq3    ACAACGTCTACTTACGTTGCATCGTCATGCTGCACTGATGATG-------------
   seq4    ACAACGTCTACTTACGTTGCATCTTCGGTCATGCTGCATTACGTAGTCTGATGATG
   ```

   or

   ```
   $ seqkit fx2tab seqs.msa.fa | cut -f 2
   ACAACGTCTACTTACGTTGCAT----CGTCATGCTGCATTACGTAGTCTGATGATG
   ---------------ACACCGTCTACTTTCATGCTGCATTACGTAGTCTGATGATG
   ACAACGTCTACTTACGTTGCATCGTCATGCTGCACTGATGATG-------------
   ACAACGTCTACTTACGTTGCATCTTCGGTCATGCTGCATTACGTAGTCTGATGATG
   ```

   For me, it's useful when 1) manually assembling Sanger sequencing result,
   2) designing site specific PCR primers.
4. Remove gaps

   ```
   $ seqkit seq seqs.msa.fa -g
   >seq1
   ACAACGTCTACTTACGTTGCATCGTCATGCTGCATTACGTAGTCTGATGATG
   >seq2
   ACACCGTCTACTTTCATGCTGCATTACGTAGTCTGATGATG
   >seq3
   ACAACGTCTACTTACGTTGCATCGTCATGCTGCACTGATGATG
   >seq4
   ACAACGTCTACTTACGTTGCATCTTCGGTCATGCTGCATTACGTAGTCTGATGATG
   ```

## Play with miRNA hairpins

### Dataset

`hairpin.fa.gz`
from The miRBase Sequence Database -- Release 21

### Quick glance

1. Sequence number

   ```
   $ seqkit stat hairpin.fa.gz
   file           format  type  num_seqs    sum_len  min_len  avg_len  max_len
   hairpin.fa.gz  FASTA   RNA     28,645  2,949,871       39      103    2,354
   ```
2. First 10 bases

   ```
   $ zcat hairpin.fa.gz | seqkit subseq -r 1:10 | seqkit sort -s | seqkit seq -s | head -n 10
   AAAAAAAAAA
   AAAAAAAAAA
   AAAAAAAAAG
   AAAAAAAAAG
   AAAAAAAAAG
   AAAAAAAAAU
   AAAAAAAAGG
   AAAAAAACAU
   AAAAAAACGA
   AAAAAAAUUA
   ```

   hmm, nothing special, non-coding RNA~

### Repeated hairpin sequences

We may want to check how may identical hairpins among different species there are.
`seqkit rmdup` could remove duplicated sequences by sequence content,
and save the replicates to another file (here is `duplicated.fa.gz`),
as well as replicating details (`duplicated.detail.txt`,
1th column is the repeated number,
2nd column contains sequence IDs seperated by comma).

```
$ seqkit rmdup -s -i hairpin.fa.gz -o clean.fa.gz -d duplicated.fa.gz -D duplicated.detail.txt

$ head -n 5 duplicated.detail.txt
18      dre-mir-430c-1, dre-mir-430c-2, dre-mir-430c-3, dre-mir-430c-4, dre-mir-430c-5, dre-mir-430c-6, dre-mir-430c-7, dre-mir-430c-8, dre-mir-430c-9, dre-mir-430c-10, dre-mir-430c-11, dre-mir-430c-12, dre-mir-430c-13, dre-mir-430c-14, dre-mir-430c-15, dre-mir-430c-16, dre-mir-430c-17, dre-mir-430c-18
16      hsa-mir-29b-2, mmu-mir-29b-2, rno-mir-29b-2, ptr-mir-29b-2, ggo-mir-29b-2, ppy-mir-29b-2, sla-mir-29b, mne-mir-29b, ppa-mir-29b-2, bta-mir-29b-2, mml-mir-29b-2, eca-mir-29b-2, aja-mir-29b, oar-mir-29b-1, oar-mir-29b-2, rno-mir-29b-3
15      dme-mir-125, dps-mir-125, dan-mir-125, der-mir-125, dgr-mir-125-1, dgr-mir-125-2, dmo-mir-125, dpe-mir-125-2, dpe-mir-125-1, dpe-mir-125-3, dse-mir-125, dsi-mir-125, dvi-mir-125, dwi-mir-125, dya-mir-125
13      hsa-mir-19b-1, ggo-mir-19b-1, age-mir-19b-1, ppa-mir-19b-1, ppy-mir-19b-1, ptr-mir-19b-1, mml-mir-19b-1, sla-mir-19b-1, lla-mir-19b-1, mne-mir-19b-1, bta-mir-19b, oar-mir-19b, chi-mir-19b
13      hsa-mir-20a, ssc-mir-20a, ggo-mir-20a, age-mir-20, ppa-mir-20, ppy-mir-20a, ptr-mir-20a, mml-mir-20a, sla-mir-20, lla-mir-20, mne-mir-20, bta-mir-20a, eca-mir-20a
```

The result shows the most conserved miRNAs among different species,
`mir-29b`, `mir-125`, `mir-19b-1` and `mir-20a`.
And the `dre-miR-430c` has the most multicopies in *Danio rerio*.

### Hairpins in different species

1. Before spliting by species, let's take a look at the sequence names.

   ```
   $ seqkit seq hairpin.fa.gz -n | head -n 3
   cel-let-7 MI0000001 Caenorhabditis elegans let-7 stem-loop
   cel-lin-4 MI0000002 Caenorhabditis elegans lin-4 stem-loop
   cel-mir-1 MI0000003 Caenorhabditis elegans miR-1 stem-loop
   ```

   The first three letters (e.g. `cel`) are the abbreviation of species names.
   So we could split hairpins by the first letters by defining custom
   sequence ID parsing regular expression `^([\w]+)\-`.

   By default, `seqkit` takes the first non-space letters as sequence ID.
   For example,

   | FASTA head | ID |
   | --- | --- |
   | >123456 gene name | 123456 |
   | >longname | longname |
   | >gi|110645304|ref|NC\_002516.2| Pseudomona | gi|110645304|ref|NC\_002516.2| |

   But for some sequences from NCBI,
   e.g. `>gi|110645304|ref|NC_002516.2| Pseudomona`, the ID is `NC_002516.2`.
   In this case, we could set sequence ID parsing regular expression by flag
   `--id-regexp "\|([^\|]+)\| "` or just use flag `--id-ncbi`. If you want
   the `gi` number, then use `--id-regexp "^gi\|([^\|]+)\|"`.
2. Split sequences by species.
   A custom ID parsing regular expression is used, `^([\w]+)\-`.

   ```
   $ seqkit split hairpin.fa.gz -i --id-regexp "^([\w]+)\-" --two-pass
   ```

   ***To reduce memory usage when splitting big file, we should always use flag `--two-pass`***
3. Species with most miRNA hairpins. Third column is the sequences number.

   ```
   $ cd hairpin.fa.gz.split/;
   $ seqkit stat hairpin.id_* | csvtk space2tab | csvtk -t sort -k num_seqs:nr | csvtk -t pretty| more
   file                     format   type   num_seqs   sum_len   min_len   avg_len   max_len
   hairpin.id_hsa.fasta     FASTA    RNA    1,881      154,242   82        82        82
   hairpin.id_mmu.fasta     FASTA    RNA    1,193      107,370   90        90        90
   hairpin.id_bta.fasta     FASTA    RNA    808        61,408    76        76        76
   hairpin.id_gga.fasta     FASTA    RNA    740        42,180    57        57        57
   hairpin.id_eca.fasta     FASTA    RNA    715        89,375    125       125       125
   hairpin.id_mtr.fasta     FASTA    RNA    672        231,840   345       345       345
   ```

   Here, a CSV/TSV tool csvtk
   is used to sort and view the result.

For human miRNA hairpins

1. Length distribution.
   `seqkit fx2tab` could show extra information like sequence length, GC content.
   A distribution ploting script is used, (
   plot\_distribution.py )

   ```
   $ seqkit fx2tab hairpin.id_hsa.fa.gz -l | cut -f 3  | plot_distribution.py -o hairpin.id_hsa.fa.gz.lendist.png
   ```

## Bacteria genome

### Dataset

Pseudomonas aeruginosa PAO1,
files:

- Genbank file `PAO1.gb`
- Genome FASTA file `PAO1.fasta`
- GTF file `PAO1.gtf` was created with `extract_features_from_genbank_file.py`, by

  ```
  extract_features_from_genbank_file.py  PAO1.gb -t . -f gtf > PAO1.gtf
  ```

### Motif distribution

Motifs

```
$ cat motifs.fa
>GTAGCGS
GTAGCGS
>GGWGKTCG
GGWGKTCG
```

1. Sliding. Remember flag `--id-ncbi`, do you?
   By the way, do not be scared by the long flag `--circle-genome`, `--step`
   and so on. They have short ones, `-c`, `-s`

   ```
   $ seqkit sliding --id-ncbi --circular-genome --step 20000 --window 200000 PAO1.fasta -o PAO1.fasta.sliding.fa

   $ seqkit stat PAO1.fasta.sliding.fa
   file                   format  type  num_seqs     sum_len  min_len  avg_len  max_len
   PAO1.fasta.sliding.fa  FASTA   DNA        314  62,800,000  200,000  200,000  200,000
   ```
2. Locating motifs

   ```
   $ seqkit locate --id-ncbi --ignore-case --degenerate --pattern-file motifs.fa  PAO1.fasta.sliding.fa -o  PAO1.fasta.sliding.fa.motifs.tsv
   ```
3. Ploting distribution (plot\_motif\_distribution.R)

   ```
   # preproccess
   $ perl -ne 'if (/_sliding:(\d+)-(\d+)\t(.+)/) {$loc= $1 + 100000; print "$loc\t$3\n";} else {print}' PAO1.fasta.sliding.fa.motifs.tsv  > PAO1.fasta.sliding.fa.motifs.tsv2

   # plot
   $ ./plot_motif_distribution.R
   ```

   Result

### Find multicopy genes

1. Get all CDS sequences

   ```
   $ seqkit subseq --id-ncbi --gtf PAO1.gtf --feature cds PAO1.fasta -o PAO1.cds.fasta

   $ seqkit stat *.fasta
   file            format  type  num_seqs    sum_len    min_len    avg_len    max_len
   PAO1.cds.fasta  FASTA   DNA      5,572  5,593,306         72    1,003.8     16,884
   PAO1.fasta      FASTA   DNA          1  6,264,404  6,264,404  6,264,404  6,264,404
   ```
2. Get duplicated sequences

   ```
   $ seqkit rmdup --by-seq --ignore-case PAO1.cds.fasta -o PAO1.cds.uniq.fasta --dup-seqs-file PAO1.cds.dup.fasta --dup-num-file PAO1.cds.dup.text

   $ cat PAO1.cds.dup.text
   6       NC_002516.2_500104:501120:-, NC_002516.2_2556948:2557964:+, NC_002516.2_3043750:3044766:-, NC_002516.2_3842274:3843290:-, NC_002516.2_4473623:4474639:+, NC_002516.2_5382796:5383812:-
   2       NC_002516.2_2073555:2075438:+, NC_002516.2_4716660:4718543:+
   2       NC_002516.2_2072935:2073558:+, NC_002516.2_4716040:4716663:+
   2       NC_002516.2_2075452:2076288:+, NC_002516.2_4718557:4719393:+
   ```

### Flanking sequences

1. Get CDS and 1000 bp upstream sequence

   ```
   $ seqkit subseq --id-ncbi --gtf PAO1.gtf --feature cds PAO1.fasta --up-stream 1000
   ```
2. Get 1000 bp upstream sequence of CDS, *NOT* including CDS.

   ```
   $ seqkit subseq --id-ncbi --gtf PAO1.gtf --feature cds PAO1.fasta --up-stream 1000 --only-flank
   ```

Please enable JavaScript to view the comments powered by Disqus.

---

Documentation built with MkDocs.

×Close

#### Search

From here you can search these documents. Enter
your search terms below.
