# Supplementary material for: SeqKit: A Cross-Platform and Ultrafast Toolkit for FASTA/Q File Manipulation
Source: PLoS One. 2016 Oct 5;11(10):e0163962. doi: 10.1371/journal.pone.0163962 (PMC5051824; doi:10.1371/journal.pone.0163962)
Supplement: S2 File — All data supporting this article including source code, documents, executable binary files, benchmark scripts and plotting scripts. (ZIP) [file pone.0163962.s002.zip › SeqKit-supplementary-data2/doc/site/usage/index.html]

Usage - SeqKit - Ultrafast FASTA/Q kit


Toggle navigation


SeqKit - Ultrafast FASTA/Q kit

- Home
- Download
- Usage
- Tutorial
- Benchmark
- YanLi Lab

- Search
- Previous
- Next
- GitHub

- Usage and Examples
- Technical details and guides for use
- seqkit
- seq
- subseq
- sliding
- stat
- fq2fa
- fx2tab & tab2fx
- grep
- locate
- rmdup
- common
- split
- sample
- head
- replace
- shuffle
- sort

# Usage and Examples

## Technical details and guides for use

### FASTA/Q format parsing

SeqKit uses author's lightweight and high-performance bioinformatics packages
bio for FASTA/Q parsing,
which has high performance
close to the
famous C lib klib (kseq.h).

### Sequence formats and types

SeqKit seamlessly support FASTA and FASTQ format.
Sequence format is automatically detected.
All subcommands except for `faidx` can handle both formats.
And only when some commands (`subseq`, `split`, `sort` and `shuffle`)
which utilise FASTA index to improve perfrmance for large files in two pass mode
(by flag `--two-pass`), only FASTA format is supported.

Sequence type (DNA/RNA/Protein) is automatically detected by leading subsequences
of the first sequences in file or STDIN. The length of the leading subsequences
is configurable by global flag `--alphabet-guess-seq-length` with default value
of 10000. If length of the sequences is less than that, whole sequences will
be checked.

### Sequence ID

By default, most softwares, including `seqkit`, take the leading non-space
letters as sequence identifier (ID). For example,

| FASTA header | ID |
| --- | --- |
| >123456 gene name | 123456 |
| >longname | longname |
| >gi|110645304|ref|NC\_002516.2| Pseudomona | gi|110645304|ref|NC\_002516.2| |

But for some sequences from NCBI,
e.g. `>gi|110645304|ref|NC_002516.2| Pseudomona`, the ID is `NC_002516.2`.
In this case, we could set sequence ID parsing regular expression by global flag
`--id-regexp "\|([^\|]+)\| "` or just use flag `--id-ncbi`. If you want
the `gi` number, then use `--id-regexp "^gi\|([^\|]+)\|"`.

### FASTA index

For some commands, including `subseq`, `split`, `sort` and `shuffle`,
when input files are (plain or gzipped) FASTA files,
FASTA index would be optional used for
rapid access of sequences and reducing memory occupation.

ATTENTION: the `.seqkit.fai` file created by SeqKit is a little different from `.fai` file
created by `samtools`. SeqKit uses full sequence head instead of just ID as key.

### Parallelization of CPU intensive jobs

The validation of sequences bases and complement process of sequences
are parallelized for large sequences.

Parsing of line-based files, including BED/GFF file and ID list file are also parallelized.

The Parallelization is implemented by multiple goroutines in golang
which are similar to but much
lighter weight than threads. The concurrency number is configurable with global
flag `-j` or `--threads` (default value: 1 for single-CPU PC, 2 for others).

### Memory occupation

Most of the subcommands do not read whole FASTA/Q records in to memory,
including `stat`, `fq2fa`, `fx2tab`, `tab2fx`, `grep`, `locate`, `replace`,
`seq`, `sliding`, `subseq`.

Note that when using `subseq --gtf | --bed`, if the GTF/BED files are too
big, the memory usage will increase.
You could use `--chr` to specify chromesomes and `--feature` to limit features.

Some subcommands need to store sequences or heads in memory, but there are
strategy to reduce memory occupation, including `rmdup` and `common`.
When comparing with sequences, MD5 digest could be used to replace sequence by
flag `-m` (`--md5`).

Some subcommands could either read all records or read the files twice by flag
`-2` (`--two-pass`), including `sample`, `split`, `shuffle` and `sort`.
They use FASTA index for rapid acccess of sequences and reducing memory occupation.

### Reproducibility

Subcommands `sample` and `shuffle` use random function, random seed could be
given by flag `-s` (`--rand-seed`). This makes sure that sampling result could be
reproduced in different environments with same random seed.

## seqkit

Usage

```
SeqKit -- a cross-platform and ultrafast toolkit for FASTA/Q file manipulation

Version: 0.3.1.1

Author: Wei Shen <shenwei356@gmail.com>

Documents  : http://shenwei356.github.io/seqkit
Source code: https://github.com/shenwei356/seqkit

Usage:
  seqkit [command]

Available Commands:
  common      find common sequences of multiple files by id/name/sequence
  faidx       create FASTA index file
  fq2fa       covert FASTQ to FASTA
  fx2tab      covert FASTA/Q to tabular format (with length/GC content/GC skew)
  grep        search sequences by pattern(s) of name or sequence motifs
  head        print first N FASTA/Q records
  locate      locate subsequences/motifs
  rename      rename duplicated IDs
  replace     replace name/sequence by regular expression
  rmdup       remove duplicated sequences by id/name/sequence
  sample      sample sequences by number or proportion
  seq         transform sequences (revserse, complement, extract ID...)
  shuffle     shuffle sequences
  sliding     sliding sequences, circular genome supported
  sort        sort sequences by id/name/sequence/length
  split       split sequences into files by id/seq region/size/parts
  stat        simple statistics of FASTA files
  subseq      get subsequences by region/gtf/bed, including flanking sequences
  tab2fx      covert tabular format to FASTA/Q format
  version     print version information and check for update

Flags:
      --alphabet-guess-seq-length int   length of sequence prefix of the first FASTA record based on which seqkit guesses the sequence type (0 for whole seq) (default 10000)
      --id-ncbi                         FASTA head is NCBI-style, e.g. >gi|110645304|ref|NC_002516.2| Pseud...
      --id-regexp string                regular expression for parsing ID (default "^([^\\s]+)\\s?")
  -w, --line-width int                  line width when outputing FASTA format (0 for no wrap) (default 60)
  -o, --out-file string                 out file ("-" for stdout, suffix .gz for gzipped out) (default "-")
      --quiet                           be quiet and do not show extra information
  -t, --seq-type string                 sequence type (dna|rna|protein|unlimit|auto) (for auto, it automatically detect by the first sequence) (default "auto")
  -j, --threads int                     number of CPUs. (default value: 1 for single-CPU PC, 2 for others) (default 2)

Use "seqkit [command] --help" for more information about a command.
```

### Datasets

Datasets from The miRBase Sequence Database -- Release 21

- `hairpin.fa.gz`
- `mature.fa.gz`
- `miRNA.diff.gz`

Human genome from ensembl
(For `seqkit subseq`)

- `Homo_sapiens.GRCh38.dna_sm.primary_assembly.fa.gz`
- `Homo_sapiens.GRCh38.84.gtf.gz`
- `Homo_sapiens.GRCh38.84.bed.gz` is converted from `Homo_sapiens.GRCh38.84.gtf.gz`
  by `gtf2bed`
  with command

  ```
  zcat Homo_sapiens.GRCh38.84.gtf.gz | gtf2bed --do-not-sort | gzip -c > Homo_sapiens.GRCh38.84.bed.gz
  ```

Only DNA and gtf/bed data of Chr1 were used:

- `chr1.fa.gz`

  ```
      seqkit grep -p 1 Homo_sapiens.GRCh38.dna_sm.primary_assembly.fa.gz -o chr1.fa.gz
  ```
- `chr1.gtf.gz`

  ```
      zcat Homo_sapiens.GRCh38.84.gtf.gz | grep -w '^1' | gzip -c > chr1.gtf.gz
  ```
- `chr1.bed.gz`

  ```
      zcat Homo_sapiens.GRCh38.84.bed.gz | grep -w '^1' | gzip -c > chr1.bed.gz
  ```

## seq

Usage

```
transform sequences (revserse, complement, extract ID...)

Usage:
  seqkit seq [flags]

Flags:
  -p, --complement                complement sequence (blank for Protein sequence)
      --dna2rna                   DNA to RNA
  -G, --gap-letter string         gap letters (default "- ")
  -l, --lower-case                print sequences in lower case
  -n, --name                      only print names
  -i, --only-id                   print ID instead of full head
  -q, --qual                      only print qualities
  -g, --remove-gaps               remove gaps
  -r, --reverse                   reverse sequence)
      --rna2dna                   RNA to DNA
  -s, --seq                       only print sequences
  -u, --upper-case                print sequences in upper case
  -v, --validate-seq              validate bases according to the alphabet
  -V, --validate-seq-length int   length of sequence to validate (0 for whole seq) (default 10000)
```

Examples

1. Read and print

   - From file:

     ```
     $ seqkit seq hairpin.fa.gz
     >cel-let-7 MI0000001 Caenorhabditis elegans let-7 stem-loop
     UACACUGUGGAUCCGGUGAGGUAGUAGGUUGUAUAGUUUGGAAUAUUACCACCGGUGAAC
     UAUGCAAUUUUCUACCUUACCGGAGACAGAACUCUUCGA

     $ seqkit seq read_1.fq.gz
     @HWI-D00523:240:HF3WGBCXX:1:1101:2574:2226 1:N:0:CTGTAG
     TGAGGAATATTGGTCAATGGGCGCGAGCCTGAACCAGCCAAGTAGCGTGAAGGATGACTG
     CCCTACGGGTTGTAAACTTCTTTTATAAAGGAATAAAGTGAGGCACGTGTGCCTTTTTGT
     ATGTACTTTATGAATAAGGATCGGCTAACTCCGTGCCAGCAGCCGCGGTAATACGGAGGA
     TCCGAGCGTTATCCGGATTTATTGGGTTTAAAGGGTGCGCAGGCGGT
     +
     HIHIIIIIHIIHGHHIHHIIIIIIIIIIIIIIIHHIIIIIHHIHIIIIIGIHIIIIHHHH
     HHGHIHIIIIIIIIIIIGHIIIIIGHIIIIHIIHIHHIIIIHIHHIIIIIIIGIIIIIII
     HIIIIIGHIIIIHIIIH?DGHEEGHIIIIIIIIIIIHIIHIIIHHIIHIHHIHCHHIIHG
     IHHHHHHH<GG?B@EHDE-BEHHHII5B@GHHF?CGEHHHDHIHIIH
     ```
   - From stdin:

     ```
     zcat hairpin.fa.gz | seqkit seq
     ```
2. Sequence types

   - By default, `seqkit seq` automatically detect the sequence type

     ```
     $ echo -e ">seq\nacgtryswkmbdhvACGTRYSWKMBDHV" | seqkit stat
     file  format  type  num_seqs  sum_len  min_len  avg_len  max_len
     -     FASTA   DNA          1       28       28       28       28

     $ echo -e ">seq\nACGUN ACGUN" | seqkit stat
     file  format  type  num_seqs  sum_len  min_len  avg_len  max_len
     -     FASTA   RNA          1       11       11       11       11

     $ echo -e ">seq\nabcdefghijklmnpqrstvwyz" | seqkit stat
     file  format  type     num_seqs  sum_len  min_len  avg_len  max_len
     -     FASTA   Protein         1       23       23       23       23

     $ echo -e "@read\nACTGCN\n+\n@IICCG" | seqkit stat
     file  format  type  num_seqs  sum_len  min_len  avg_len  max_len
     -     FASTQ   DNA          1        6        6        6        6
     ```
   - You can also set sequence type by flag `-t` (`--seq-type`).
     But this only take effect on subcommands `seq` and `locate`.

     ```
     $ echo -e ">seq\nabcdefghijklmnpqrstvwyz" | seqkit seq -t dna
     [INFO] when flag -t (--seq-type) given, flag -v (--validate-seq) is automatically switched on
     [ERRO] error when parsing seq: seq (invalid DNAredundant letter: e)
     ```
3. Only print names

   - Full name:

     ```
     $ seqkit seq hairpin.fa.gz -n
     cel-let-7 MI0000001 Caenorhabditis elegans let-7 stem-loop
     cel-lin-4 MI0000002 Caenorhabditis elegans lin-4 stem-loop
     cel-mir-1 MI0000003 Caenorhabditis elegans miR-1 stem-loop
     ```
   - Only ID:

     ```
     $ seqkit seq hairpin.fa.gz -n -i
     cel-let-7
     cel-lin-4
     cel-mir-1
     ```
   - Custom ID region by regular expression (this could be applied to all subcommands):

     ```
     $ seqkit seq hairpin.fa.gz -n -i --id-regexp "^[^\s]+\s([^\s]+)\s"
     MI0000001
     MI0000002
     MI0000003
     ```
4. Only print seq (global flag `-w` defines the output line width, 0 for no wrap)

   ```
   $ seqkit seq hairpin.fa.gz -s -w 0
   UACACUGUGGAUCCGGUGAGGUAGUAGGUUGUAUAGUUUGGAAUAUUACCACCGGUGAACUAUGCAAUUUUCUACCUUACCGGAGACAGAACUCUUCGA
   AUGCUUCCGGCCUGUUCCCUGAGACCUCAAGUGUGAGUGUACUAUUGAUGCUUCACACCUGGGCUCUCCGGGUACCAGGACGGUUUGAGCAGAU
   AAAGUGACCGUACCGAGCUGCAUACUUCCUUACAUGCCCAUACUAUAUCAUAAAUGGAUAUGGAAUGUAAAGAAGUAUGUAGAACGGGGUGGUAGU
   ```
5. Convert multi-line FASTQ to 4-line FASTQ

   ```
   $ seqkit seq reads_1.fq.gz -w 0
   ```
6. Reverse comlement sequence

   ```
   $ seqkit seq hairpin.fa.gz -r -p
   >cel-let-7 MI0000001 Caenorhabditis elegans let-7 stem-loop
   UCGAAGAGUUCUGUCUCCGGUAAGGUAGAAAAUUGCAUAGUUCACCGGUGGUAAUAUUCC
   AAACUAUACAACCUACUACCUCACCGGAUCCACAGUGUA
   ```
7. Remove gaps and to lower/upper case

   ```
   $ echo -e ">seq\nACGT-ACTGC-ACC" | seqkit seq -g -u
   >seq
   ACGTACTGCACC
   ```
8. RNA to DNA

   ```
   $ echo -e ">seq\nUCAUAUGCUUGUCUCAAAGAUUA" | seqkit seq --rna2dna
   >seq
   TCATATGCTTGTCTCAAAGATTA
   ```

## subseq

Usage

```
get subsequences by region/gtf/bed, including flanking sequences.

Recommendation: use plain FASTA file, so seqkit could utilize FASTA index.

The definition of region is 1-based and with some custom design.

Examples:

 1-based index    1 2 3 4 5 6 7 8 9 10
negative index    0-9-8-7-6-5-4-3-2-1
           seq    A C G T N a c g t n
           1:1    A
           2:4      C G T
         -4:-2                c g t
         -4:-1                c g t n
         -1:-1                      n
          2:-2      C G T N a c g t
          1:-1    A C G T N a c g t n

Usage:
  seqkit subseq [flags]

Flags:
      --bed string        by BED file
      --chr value         select limited sequence with sequence IDs (multiple value supported, case ignored) (default [])
  -d, --down-stream int   down stream length
      --feature value     select limited feature types (multiple value supported, case ignored, only works with GTF) (default [])
      --gtf string        by GTF (version 2.2) file
  -f, --only-flank        only return up/down stream sequence
  -r, --region string     by region. e.g 1:12 for first 12 bases, -12:-1 for last 12 bases, 13:-1 for cutting first 12 bases. type "seqkit subseq -h" for more examples
  -u, --up-stream int     up stream length
```

Examples

***Recommendation: use plain FASTA file, so seqkit could utilize FASTA index.***

1. First 12 bases

   ```
   $ zcat hairpin.fa.gz | seqkit subseq -r 1:12
   ```
2. Last 12 bases

   ```
   $ zcat hairpin.fa.gz | seqkit subseq -r -12:-1
   ```
3. Subsequences without first and last 12 bases

   ```
   $ zcat hairpin.fa.gz | seqkit subseq -r 13:-13
   ```
4. Get subsequence by GTF file

   ```
   $ cat t.fa
   >seq
   actgACTGactgn
   $ cat t.gtf
   seq     test    CDS     5       8       .       .       .       gene_id "A"; transcript_id "";
   seq     test    CDS     5       8       .       -       .       gene_id "B"; transcript_id "";
   $ seqkit

   $ seqkit subseq --gtf t.gtf t.fa
   >seq_5:8:. A
   ACTG
   >seq_5:8:- B
   CAGT
   ```

   Human genome example:

   ***AVOID loading all data from Homo\_sapiens.GRCh38.84.gtf.gz,
   the uncompressed data are so big and may exhaust your RAM.***

   We could specify chromesomes and features.

   ```
   $ seqkit subseq --gtf Homo_sapiens.GRCh38.84.gtf.gz --chr 1 --feature cds  hsa.fa > chr1.gtf.cds.fa

   $ seqkit stat chr1.gtf.cds.fa
   file             format  type  num_seqs    sum_len  min_len  avg_len  max_len
   chr1.gtf.cds.fa  FASTA   DNA     65,012  9,842,274        1    151.4   12,045
   ```
5. Get CDS and 3bp up-stream sequences

   ```
   $ seqkit subseq --gtf t.gtf t.fa -u 3
   >seq_5:8:._us:3 A
   ctgACTG
   >seq_5:8:-_us:3 B
   agtCAGT
   ```
6. Get 3bp up-stream sequences of CDS, not including CDS

   ```
   $ seqkit subseq --gtf t.gtf t.fa -u 3 -f
   >seq_5:8:._usf:3 A
   ctg
   >seq_5:8:-_usf:3 B
   agt
   ```
7. Get subsequences by BED file.

   ***AVOID loading all data from Homo\_sapiens.GRCh38.84.gtf.gz,
   the uncompressed data are so big and may exhaust your RAM.***

   ```
   $  seqkit subseq --bed Homo_sapiens.GRCh38.84.bed.gz --chr 1 hsa.fa >  chr1.bed.gz.fa
   ```

   We may need to remove duplicated sequences

   ```
   $ seqkit subseq --bed Homo_sapiens.GRCh38.84.bed.gz --chr 1 hsa.fa | seqkit rmdup > chr1.bed.rmdup.fa
   [INFO] 141060 duplicated records removed
   ```

   Summary:

   ```
   $ seqkit stat chr1.gz.*.gz
   file               seq_format   seq_type   num_seqs   min_len   avg_len     max_len
   chr1.gz.fa         FASTA        DNA         231,974         1   3,089.5   1,551,957
   chr1.gz.rmdup.fa   FASTA        DNA          90,914         1   6,455.8   1,551,957
   ```

## sliding

Usage

```
sliding sequences, circular genome supported

Usage:
  seqkit sliding [flags]

Flags:
  -C, --circular-genome   circular genome
  -s, --step int        step size
  -W, --window int      window size
```

Examples

1. General use

   ```
   $ echo -e ">seq\nACGTacgtNN" | seqkit sliding -s 3 -W 6
   >seq_sliding:1-6
   ACGTac
   >seq_sliding:4-9
   TacgtN
   ```
2. Circular genome

   ```
   $ echo -e ">seq\nACGTacgtNN" | seqkit sliding -s 3 -W 6 -C
   >seq_sliding:1-6
   ACGTac
   >seq_sliding:4-9
   TacgtN
   >seq_sliding:7-2
   gtNNAC
   >seq_sliding:10-5
   NACGTa
   ```
3. Generate GC content for ploting

   ```
   $ zcat hairpin.fa.gz | seqkit fx2tab | head -n 1 | seqkit tab2fx | seqkit sliding -s 5 -W 30 | seqkit fx2tab -n -g
   cel-let-7_sliding:1-30          50.00
   cel-let-7_sliding:6-35          46.67
   cel-let-7_sliding:11-40         43.33
   cel-let-7_sliding:16-45         36.67
   cel-let-7_sliding:21-50         33.33
   cel-let-7_sliding:26-55         40.00
   ...
   ```

## stat

Usage

```
simple statistics of FASTA files

Usage:
  seqkit stat [flags]
```

Eexamples

1. General use

   ```
   $ seqkit stat *.f{a,q}.gz
   file           format  type  num_seqs    sum_len  min_len  avg_len  max_len
   hairpin.fa.gz  FASTA   RNA     28,645  2,949,871       39      103    2,354
   mature.fa.gz   FASTA   RNA     35,828    781,222       15     21.8       34
   reads_1.fq.gz  FASTQ   DNA      2,500    567,516      226      227      229
   reads_2.fq.gz  FASTQ   DNA      2,500    560,002      223      224      225
   ```

## fq2fa

Usage

```
covert FASTQ to FASTA

Usage:
  seqkit fq2fa [flags]
```

Examples

```
seqkit fq2fa reads_1.fq.gz -o reads1_.fa.gz
```

## fx2tab & tab2fx

Usage (fx2tab)

```
covert FASTA/Q to tabular format, and provide various information,
like sequence length, GC content/GC skew.

Usage:
  seqkit fx2tab [flags]

Flags:
  -B, --base-content value   print base content. (case ignored, multiple values supported) e.g. -B AT -B N (default [])
  -g, --gc                   print GC content
  -G, --gc-skew              print GC-Skew
  -H, --header-line          print header line
  -l, --length               print sequence length
  -n, --name                 only print names (no sequences and qualities)
  -i, --only-id              print ID instead of full head
```

Usage (tab2fx)

```
covert tabular format (first two/three columns) to FASTA/Q format

Usage:
  seqkit tab2fx [flags]

Flags:
  -p, --comment-line-prefix value   comment line prefix (default [#,//])
```

Examples

1. Default output

   ```
   $ seqkit fx2tab hairpin.fa.gz | head -n 2
   cel-let-7 MI0000001 Caenorhabditis elegans let-7 stem-loop      UACACUGUGGAUCCGGUGAGGUAGUAGGUUGUAUAGUUUGGAAUAUUACCACCGGUGAACUAUGCAAUUUUCUACCUUACCGGAGACAGAACUCUUCGA
   cel-lin-4 MI0000002 Caenorhabditis elegans lin-4 stem-loop      AUGCUUCCGGCCUGUUCCCUGAGACCUCAAGUGUGAGUGUACUAUUGAUGCUUCACACCUGGGCUCUCCGGGUACCAGGACGGUUUGAGCAGAU
   ```
2. Print sequence length, GC content, and only print names (no sequences),
   we could also print title line by flag `-T`.

   ```
   $ seqkit fx2tab hairpin.fa.gz -l -g -n -i -H | head -n 4 | csvtk -t -C '&' pretty
   #name       seq   qual   length   GC
   cel-let-7                99       43.43
   cel-lin-4                94       54.26
   cel-mir-1                96       40.62
   ```
3. Use fx2tab and tab2fx in pipe

   ```
   $ zcat hairpin.fa.gz | seqkit fx2tab | seqkit tab2fx

   $ zcat reads_1.fq.gz | seqkit fx2tab | seqkit tab2fx
   ```
4. Sort sequences by length (use `seqkit sort -l`)

   ```
   $ zcat hairpin.fa.gz | seqkit fx2tab -l | sort -t"`echo -e '\t'`" -n -k4,4 | seqkit tab2fx
   >cin-mir-4129 MI0015684 Ciona intestinalis miR-4129 stem-loop
   UUCGUUAUUGGAAGACCUUAGUCCGUUAAUAAAGGCAUC
   >mmu-mir-7228 MI0023723 Mus musculus miR-7228 stem-loop
   UGGCGACCUGAACAGAUGUCGCAGUGUUCGGUCUCCAGU
   >cin-mir-4103 MI0015657 Ciona intestinalis miR-4103 stem-loop
   ACCACGGGUCUGUGACGUAGCAGCGCUGCGGGUCCGCUGU

   $ seqkit sort -l hairpin.fa.gz
   ```

   Sorting or filtering by GC (or other base by -flag `-B`) content could also achieved in similar way.
5. Get first 1000 sequences

   ```
   $ seqkit fx2tab hairpin.fa.gz | head -n 1000 | seqkit tab2fx

   $ seqkit fx2tab reads_1.fq.gz | head -n 1000 | seqkit tab2fx
   ```

**Extension**

After converting FASTA to tabular format with `seqkit fx2tab`,
it could be handled with CSV/TSV tools,
e.g. csvtk, a cross-platform, efficient and practical CSV/TSV toolkit

- `csvtk grep` could be used to filter sequences (similar with `seqkit grep`)
- `csvtk inter`
  computates intersection of multiple files. It could achieve similar function
  as `seqkit common -n` along with shell.
- `csvtk join` joins multiple CSV/TSV files by multiple IDs.
- csv\_melt
  provides melt function, could be used in preparation of data for ploting.

## grep

Usage

```
search sequences by pattern(s) of name or sequence motifs

Usage:
  seqkit grep [flags]

Flags:
  -n, --by-name               match by full name instead of just id
  -s, --by-seq                match by seq
  -d, --degenerate            pattern/motif contains degenerate base
      --delete-matched        delete matched pattern to speedup
  -i, --ignore-case           ignore case
  -v, --invert-match          invert the sense of matching, to select non-matching records
  -p, --pattern value         search pattern (multiple values supported) (default [])
  -f, --pattern-file string   pattern file
  -r, --use-regexp            patterns are regular expression
```

Examples

1. Extract human hairpins (i.e. sequences with name starting with `hsa`)

   ```
   $ zcat hairpin.fa.gz | seqkit grep -r -p ^hsa
   >hsa-let-7a-1 MI0000060 Homo sapiens let-7a-1 stem-loop
   UGGGAUGAGGUAGUAGGUUGUAUAGUUUUAGGGUCACACCCACCACUGGGAGAUAACUAU
   ACAAUCUACUGUCUUUCCUA
   >hsa-let-7a-2 MI0000061 Homo sapiens let-7a-2 stem-loop
   AGGUUGAGGUAGUAGGUUGUAUAGUUUAGAAUUACAUCAAGGGAGAUAACUGUACAGCCU
   CCUAGCUUUCCU
   ```
2. Remove human and mice hairpins.

   ```
   $ zcat hairpin.fa.gz | seqkit grep -r -p ^hsa -p ^mmu -v
   ```
3. Extract new entries by information from miRNA.diff.gz

   1. Get IDs of new entries.

      ```
      $ zcat miRNA.diff.gz | grep ^# -v | grep NEW | cut -f 2 > list
      $ more list
      cfa-mir-486
      cfa-mir-339-1
      pmi-let-7
      ```
   2. Extract by ID list file

      ```
      $ zcat hairpin.fa.gz | seqkit grep -f list > new.fa
      ```
4. Extract sequences starting with AGGCG

   ```
   $ zcat hairpin.fa.gz | seqkit grep -s -r -i -p ^aggcg
   ```
5. Extract sequences with TTSAA (AgsI digest site) in SEQUENCE. Base S stands for C or G.

   ```
   $ zcat hairpin.fa.gz | seqkit grep -s -d -i -p TTSAA
   ```

   It's equal to but simpler than:

   ```
   $ zcat hairpin.fa.gz | seqkit grep -s -r -i -p TT[CG]AA
   ```

## locate

Usage

```
locate subsequences/motifs

Motifs could be EITHER plain sequence containing "ACTGN" OR regular
expression like "A[TU]G(?:.{3})+?[TU](?:AG|AA|GA)" for ORFs.
Degenerate bases like "RYMM.." are also supported by flag -d.

By default, motifs are treated as regular expression.
When flag -d given, regular expression may be wrong.
For example: "\w" will be wrongly converted to "\[AT]".

Usage:
  seqkit locate [flags]

Flags:
  -d, --degenerate                pattern/motif contains degenerate base
  -i, --ignore-case               ignore case
  -P, --only-positive-strand      only search at positive strand
  -p, --pattern value             search pattern/motif (multiple values supported) (default [])
  -f, --pattern-file string       pattern/motif file (FASTA format)
  -V, --validate-seq-length int   length of sequence to validate (0 for whole seq) (default 10000)
```

Examples

1. Locate ORFs.

   ```
   $ zcat hairpin.fa.gz | seqkit locate -i -p "A[TU]G(?:.{3})+?[TU](?:AG|AA|GA)"
   seqID   patternName     pattern strand  start   end     matched
   cel-lin-4       A[TU]G(?:.{3})+?[TU](?:AG|AA|GA)        A[TU]G(?:.{3})+?[TU](?:AG|AA|GA)        +  136      AUGCUUCCGGCCUGUUCCCUGAGACCUCAAGUGUGA
   cel-mir-1       A[TU]G(?:.{3})+?[TU](?:AG|AA|GA)        A[TU]G(?:.{3})+?[TU](?:AG|AA|GA)        +  54       95      AUGGAUAUGGAAUGUAAAGAAGUAUGUAGAACGGGGUGGUAG
   cel-mir-1       A[TU]G(?:.{3})+?[TU](?:AG|AA|GA)        A[TU]G(?:.{3})+?[TU](?:AG|AA|GA)        -  43       51      AUGAUAUAG
   ```
2. Locate Motif.

   ```
   $ zcat hairpin.fa.gz | seqkit locate -i -d -p AUGGACUN
   seqID         patternName   pattern    strand   start   end   matched
   cel-mir-58a   AUGGACUN      AUGGACUN   +        81      88    AUGGACUG
   ath-MIR163    AUGGACUN      AUGGACUN   -        122     129   AUGGACUC
   ```

   Notice that `seqkit grep` only searches in positive strand, but `seqkit loate` could recognize both strand

## rmdup

Usage

```
remove duplicated sequences by id/name/sequence

Usage:
  seqkit rmdup [flags]

Flags:
    -n, --by-name                by full name instead of just id
    -s, --by-seq                 by seq
    -D, --dup-num-file string    file to save number and list of duplicated seqs
    -d, --dup-seqs-file string   file to save duplicated seqs
    -i, --ignore-case            ignore case
    -m, --md5                    use MD5 instead of original seqs to reduce memory usage when comparing by seqs
```

Examples

Similar to `common`.

1. General use

   ```
   $ zcat hairpin.fa.gz | seqkit rmdup -s -o clean.fa.gz
   [INFO] 2226 duplicated records removed

   $ zcat reads_1.fq.gz | seqkit rmdup -s -o clean.fa.gz
   [INFO] 1086 duplicated records removed
   ```
2. Save duplicated sequences to file

   ```
   $ zcat hairpin.fa.gz | seqkit rmdup -s -i -m -o clean.fa.gz -d duplicated.fa.gz -D duplicated.detail.txt

   $ cat duplicated.detail.txt   # here is not the entire list
   3   hsa-mir-424, mml-mir-424, ppy-mir-424
   3   hsa-mir-342, mml-mir-342, ppy-mir-342
   2   ngi-mir-932, nlo-mir-932
   2   ssc-mir-9784-1, ssc-mir-9784-2
   ```

## common

Usage

```
find common sequences of multiple files by id/name/sequence

Usage:
  seqkit common [flags]

Flags:
    -n, --by-name       match by full name instead of just id
    -s, --by-seq        match by sequence
    -i, --ignore-case   ignore case
    -m, --md5           use MD5 instead of original seqs to reduce memory usage when comparing by seqs
```

Examples

1. By ID (default)

   ```
   seqkit common file*.fa -o common.fasta
   ```
2. By full name

   ```
   seqkit common file*.fa -n -o common.fasta
   ```
3. By sequence

   ```
   seqkit common file*.fa -s -i -o common.fasta
   ```
4. By sequence (***for large sequences***)

   ```
   seqkit common file*.fa -s -i -o common.fasta --md5
   ```

## split

Usage

```
split sequences into files by name ID, subsequence of given region,
part size or number of parts.

The definition of region is 1-based and with some custom design.

Examples:

 1-based index    1 2 3 4 5 6 7 8 9 10
negative index    0-9-8-7-6-5-4-3-2-1
           seq    A C G T N a c g t n
           1:1    A
           2:4      C G T
         -4:-2                c g t
         -4:-1                c g t n
         -1:-1                      n
          2:-2      C G T N a c g t
          1:-1    A C G T N a c g t n

Usage:
  seqkit split [flags]

Flags:
Flags:
  -i, --by-id              split squences according to sequence ID
  -p, --by-part int        split squences into N parts
  -r, --by-region string   split squences according to subsequence of given region. e.g 1:12 for first 12 bases, -12:-1 for last 12 bases. type "seqkit split -h" for more examples
  -s, --by-size int        split squences into multi parts with N sequences
  -d, --dry-run            dry run, just print message and no files will be created.
  -f, --force              overwrite output directory
  -k, --keep-temp          keep tempory FASTA and .fai file when using 2-pass mode
  -m, --md5                use MD5 instead of region sequence in output file when using flag -r (--by-region)
  -O, --out-dir string     output directory (default value is infile.split)
  -2, --two-pass           two-pass mode read files twice to lower memory usage. (only for FASTA format)
```

Examples

1. Split sequences into parts with at most 10000 sequences

   ```
   $ seqkit split hairpin.fa.gz -s 10000
   [INFO] split into 10000 seqs per file
   [INFO] write 10000 sequences to file: hairpin.fa.part_001.gz
   [INFO] write 10000 sequences to file: hairpin.fa.part_002.gz
   [INFO] write 8645 sequences to file: hairpin.fa.part_003.gz
   ```
2. Split sequences into 4 parts

   ```
   $ seqkit split hairpin.fa.gz -p 4
   [INFO] split into 4 parts
   [INFO] read sequences ...
   [INFO] read 28645 sequences
   [INFO] write 7162 sequences to file: hairpin.fa.part_001.gz
   [INFO] write 7162 sequences to file: hairpin.fa.part_002.gz
   [INFO] write 7162 sequences to file: hairpin.fa.part_003.gz
   [INFO] write 7159 sequences to file: hairpin.fa.part_004.gz
   ```

   ***To reduce memory usage when spliting big file, we should alwasy use flag `--two-pass`***

   ```
   $ seqkit split hairpin.fa.gz -p 4 -2
   [INFO] split into 4 parts
   [INFO] read and write sequences to tempory file: hairpin.fa.gz.fa ...
   [INFO] create and read FASTA index ...
   [INFO] read sequence IDs from FASTA index ...
   [INFO] 28645 sequences loaded
   [INFO] write 7162 sequences to file: hairpin.part_001.fa.gz
   [INFO] write 7162 sequences to file: hairpin.part_002.fa.gz
   [INFO] write 7162 sequences to file: hairpin.part_003.fa.gz
   [INFO] write 7159 sequences to file: hairpin.part_004.fa.gz
   ```
3. Split sequences by species. i.e. by custom IDs (first three letters)

   ```
   $ seqkit split hairpin.fa.gz -i --id-regexp "^([\w]+)\-" -2
   [INFO] split by ID. idRegexp: ^([\w]+)\-
   [INFO] read and write sequences to tempory file: hairpin.fa.gz.fa ...
   [INFO] create and read FASTA index ...
   [INFO] create FASTA index for hairpin.fa.gz.fa
   [INFO] read sequence IDs from FASTA index ...
   [INFO] 28645 sequences loaded
   [INFO] write 48 sequences to file: hairpin.id_cca.fa.gz
   [INFO] write 3 sequences to file: hairpin.id_hci.fa.gz
   [INFO] write 106 sequences to file: hairpin.id_str.fa.gz
   [INFO] write 1 sequences to file: hairpin.id_bkv.fa.gz
   ...
   ```
4. Split sequences by sequence region (for example, sequence barcode)

   ```
   $ seqkit split hairpin.fa.gz -r 1:3 -2
   [INFO] split by region: 1:3
   [INFO] read and write sequences to tempory file: hairpin.fa.gz.fa ...
   [INFO] read sequence IDs and sequence region from FASTA file ...
   [INFO] create and read FASTA index ...
   [INFO] write 463 sequences to file: hairpin.region_1:3_AUG.fa.gz
   [INFO] write 349 sequences to file: hairpin.region_1:3_ACU.fa.gz
   [INFO] write 311 sequences to file: hairpin.region_1:3_CGG.fa.gz
   ```

   **If region is too long, we could use falg `--md5`**,
   i.e. use MD5 instead of region sequence in output file.

   Sequence suffix could be defined as `-r -12:-1`

## sample

Usage

```
sample sequences by number or proportion.

Usage:
  seqkit sample [flags]

Flags:
  -n, --number int         sample by number (result may not exactly match)
  -p, --proportion float   sample by proportion
  -s, --rand-seed int      rand seed for shuffle (default 11)
  -2, --two-pass           2-pass mode read files twice to lower memory usage. Not allowed when reading from stdin
```

Examples

1. Sample by proportion

   ```
   $ zcat hairpin.fa.gz | seqkit sample -p 0.1 -o sample.fa.gz
   [INFO] sample by proportion
   [INFO] 2814 sequences outputed
   ```
2. Sample by number

   ```
   $ zcat hairpin.fa.gz | seqkit sample -n 1000 -o sample.fa.gz
   [INFO] sample by number
   [INFO] 949 sequences outputed
   ```

   ***To reduce memory usage when spliting big file, we could use flag `--two-pass`***

   ***We can also use `seqkit sample -p` followed with `seqkit head -n`:***

   ```
   $ zcat hairpin.fa.gz | seqkit sample -p 0.1 | seqkit head -n 1000 -o sample.fa.gz
   ```
3. Set rand seed to reproduce the result

   ```
   $ zcat hairpin.fa.gz | seqkit sample -p 0.1 -s 11
   ```
4. Most of the time, we could shuffle after sampling

   ```
   $ zcat hairpin.fa.gz | seqkit sample -p 0.1 | seqkit shuffle -o sample.fa.gz
   ```

Note that when sampling on FASTQ files, make sure using same random seed by
flag `-s` (`--rand-seed`)

## head

Usage

```
print first N FASTA/Q records

Usage:
  seqkit head [flags]

Flags:
  -n, --number int   print first N FASTA/Q records (default 10)
```

Examples

1. FASTA

   ```
   $ seqkit head -n 1 hairpin.fa.gz
   >cel-let-7 MI0000001 Caenorhabditis elegans let-7 stem-loop
   UACACUGUGGAUCCGGUGAGGUAGUAGGUUGUAUAGUUUGGAAUAUUACCACCGGUGAAC
   UAUGCAAUUUUCUACCUUACCGGAGACAGAACUCUUCGA
   ```
2. FASTQ

   ```
   $ seqkit head -n 1 reads_1.fq.gz
   @HWI-D00523:240:HF3WGBCXX:1:1101:2574:2226 1:N:0:CTGTAG
   TGAGGAATATTGGTCAATGGGCGCGAGCCTGAACCAGCCAAGTAGCGTGAAGGATGACTG
   CCCTACGGGTTGTAAACTTCTTTTATAAAGGAATAAAGTGAGGCACGTGTGCCTTTTTGT
   ATGTACTTTATGAATAAGGATCGGCTAACTCCGTGCCAGCAGCCGCGGTAATACGGAGGA
   TCCGAGCGTTATCCGGATTTATTGGGTTTAAAGGGTGCGCAGGCGGT
   +
   HIHIIIIIHIIHGHHIHHIIIIIIIIIIIIIIIHHIIIIIHHIHIIIIIGIHIIIIHHHH
   HHGHIHIIIIIIIIIIIGHIIIIIGHIIIIHIIHIHHIIIIHIHHIIIIIIIGIIIIIII
   HIIIIIGHIIIIHIIIH?DGHEEGHIIIIIIIIIIIHIIHIIIHHIIHIHHIHCHHIIHG
   IHHHHHHH<GG?B@EHDE-BEHHHII5B@GHHF?CGEHHHDHIHIIH
   ```

## replace

Usage

```
replace name/sequence/by regular expression.

Note that the replacement supports capture variables.
e.g. $1 represents the text of the first submatch.
ATTENTION: use SINGLE quote NOT double quotes in *nix OS.

Examples: Adding space to all bases.

    seqkit replace -p "(.)" -r '$1 ' -s

Or use the \ escape character.

    seqkit replace -p "(.)" -r "\$1 " -s

more on: http://shenwei356.github.io/seqkit/usage/#replace

Usage:
  seqkit replace [flags]

Flags:
  -s, --by-seq               replace seq
  -i, --ignore-case          ignore case
  -p, --pattern string       search regular expression
  -r, --replacement string   replacement. supporting capture variables.  e.g. $1 represents the text of the first submatch. ATTENTION: use SINGLE quote NOT double quotes in *nix OS or use the \ escape character. record number is also supported by "{NR}"
```

Examples

1. Remove descriptions

   ```
   $ echo -e ">seq1 abc-123\nACGT-ACGT" | seqkit replace -p " .+"
   >seq1
   ACGT-ACGT
   ```
2. Replace "-" with "="

   ```
   $ echo -e ">seq1 abc-123\nACGT-ACGT" | seqkit replace -p "\-" -r '='
   >seq1 abc=123
   ACGT-ACGT
   ```
3. Remove gaps in sequences.

   ```
   $ echo -e ">seq1 abc-123\nACGT-ACGT" | seqkit replace -p " |-" -s
   >seq1 abc-123
   ACGTACGT
   ```
4. Add space to every base. **ATTENTION: use SINGLE quote NOT double quotes in \*nix OS**

   ```
   $ echo -e ">seq1 abc-123\nACGT-ACGT" | seqkit replace -p "(.)" -r '$1 ' -s
   >seq1 abc-123
   A C G T - A C G T
   ```
5. Transpose sequence with csvtk

   ```
   $ echo -e ">seq1\nACTGACGT\n>seq2\nactgccgt" | seqkit replace -p "(.)" -r     "\$1 " -s | seqkit seq -s -u | csvtk space2tab | csvtk -t transpose
   A       A
   C       C
   T       T
   G       G
   A       C
   C       C
   G       G
   T       T
   ```
6. Rename with number of record

   ```
   echo -e ">abc\nACTG\n>123\nATTT" |  seqkit replace -p .+ -r "seq_{NR}"
   >seq_1
   ACTG
   >seq_2
   ATTT
   ```

## shuffle

Usage

```
shuffle sequences.

By default, all records will be readed into memory.
For FASTA format, use flag -2 (--two-pass) to reduce memory usage. FASTQ not
supported.

Firstly, seqkit reads the sequence IDs. If the file is not plain FASTA file,
seqkit will write the sequences to tempory files, and create FASTA index.

Secondly, seqkit shuffles sequence IDs and extract sequences by FASTA index.

Usage:
  seqkit shuffle [flags]

Flags:
  -k, --keep-temp       keep tempory FASTA and .fai file when using 2-pass mode
  -s, --rand-seed int   rand seed for shuffle (default 23)
  -2, --two-pass        two-pass mode read files twice to lower memory usage. (only for FASTA format)
```

Examples

1. General use.

   ```
   $ seqkit shuffle hairpin.fa.gz > shuffled.fa
   [INFO] read sequences ...
   [INFO] 28645 sequences loaded
   [INFO] shuffle ...
   [INFO] output ...
   ```
2. ***For big genome, you'd better use two-pass mode*** so seqkit could use
   FASTA index to reduce memory usage

   ```
   $ time seqkit shuffle -2 hsa.fa > shuffle.fa
   [INFO] create and read FASTA index ...
   [INFO] create FASTA index for hsa.fa
   [INFO] read sequence IDs from FASTA index ...
   [INFO] 194 sequences loaded
   [INFO] shuffle ...
   [INFO] output ...

   real    0m35.080s
   user    0m45.521s
   sys     0m3.411s
   ```

Note that when sampling on FASTQ files, make sure using same random seed by
flag `-s` (`--rand-seed`) for read 1 and 2 files.

## sort

Usage

```
sort sequences by id/name/sequence/length.

By default, all records will be readed into memory.
For FASTA format, use flag -2 (--two-pass) to reduce memory usage. FASTQ not
supported.

Firstly, seqkit reads the sequence head and length information.
If the file is not plain FASTA file,
seqkit will write the sequences to tempory files, and create FASTA index.

Secondly, seqkit sort sequence by head and length information
and extract sequences by FASTA index.

Usage:
  seqkit sort [flags]

Flags:
  -l, --by-length               by sequence length
  -n, --by-name                 by full name instead of just id
  -s, --by-seq                  by sequence
  -i, --ignore-case             ignore case
  -k, --keep-temp               keep tempory FASTA and .fai file when using 2-pass mode
  -r, --reverse                 reverse the result
  -L, --seq-prefix-length int   length of sequence prefix on which seqkit sorts by sequences (0 for whole sequence) (default 10000)
  -2, --two-pass                two-pass mode read files twice to lower memory usage. (only for FASTA format)
```

Examples

***For FASTA format, use flag -2 (--two-pass) to reduce memory usage***

1. sort by ID

   ```
   $ echo -e ">seq1\nACGTNcccc\n>SEQ2\nacgtnAAAA" | seqkit sort --quiet
   >SEQ2
   acgtnAAAA
   >seq1
   ACGTNcccc
   ```
2. sort by ID, ignoring case.

   ```
   $ echo -e ">seq1\nACGTNcccc\n>SEQ2\nacgtnAAAA" | seqkit sort --quiet -i
   >seq1
   ACGTNcccc
   >SEQ2
   acgtnAAAA
   ```
3. sort by seq, ignoring case.

   ```
   $ echo -e ">seq1\nACGTNcccc\n>SEQ2\nacgtnAAAA" | seqkit sort --quiet -s -i
   >SEQ2
   acgtnAAAA
   >seq1
   ACGTNcccc
   ```
4. sort by sequence length

   ```
   $ echo -e ">seq1\nACGTNcccc\n>SEQ2\nacgtnAAAAnnn\n>seq3\nacgt" | seqkit sort --quiet -l
   >seq3
   acgt
   >seq1
   ACGTNcccc
   >SEQ2
   acgtnAAAAnnn
   ```

Please enable JavaScript to view the comments powered by Disqus.

---

Documentation built with MkDocs.

×Close

#### Search

From here you can search these documents. Enter
your search terms below.
