# Supplementary figures and images for: SeqKit: A Cross-Platform and Ultrafast Toolkit for FASTA/Q File Manipulation
Source: PLoS One. 2016 Oct 5;11(10):e0163962. doi: 10.1371/journal.pone.0163962 (PMC5051824; doi:10.1371/journal.pone.0163962)

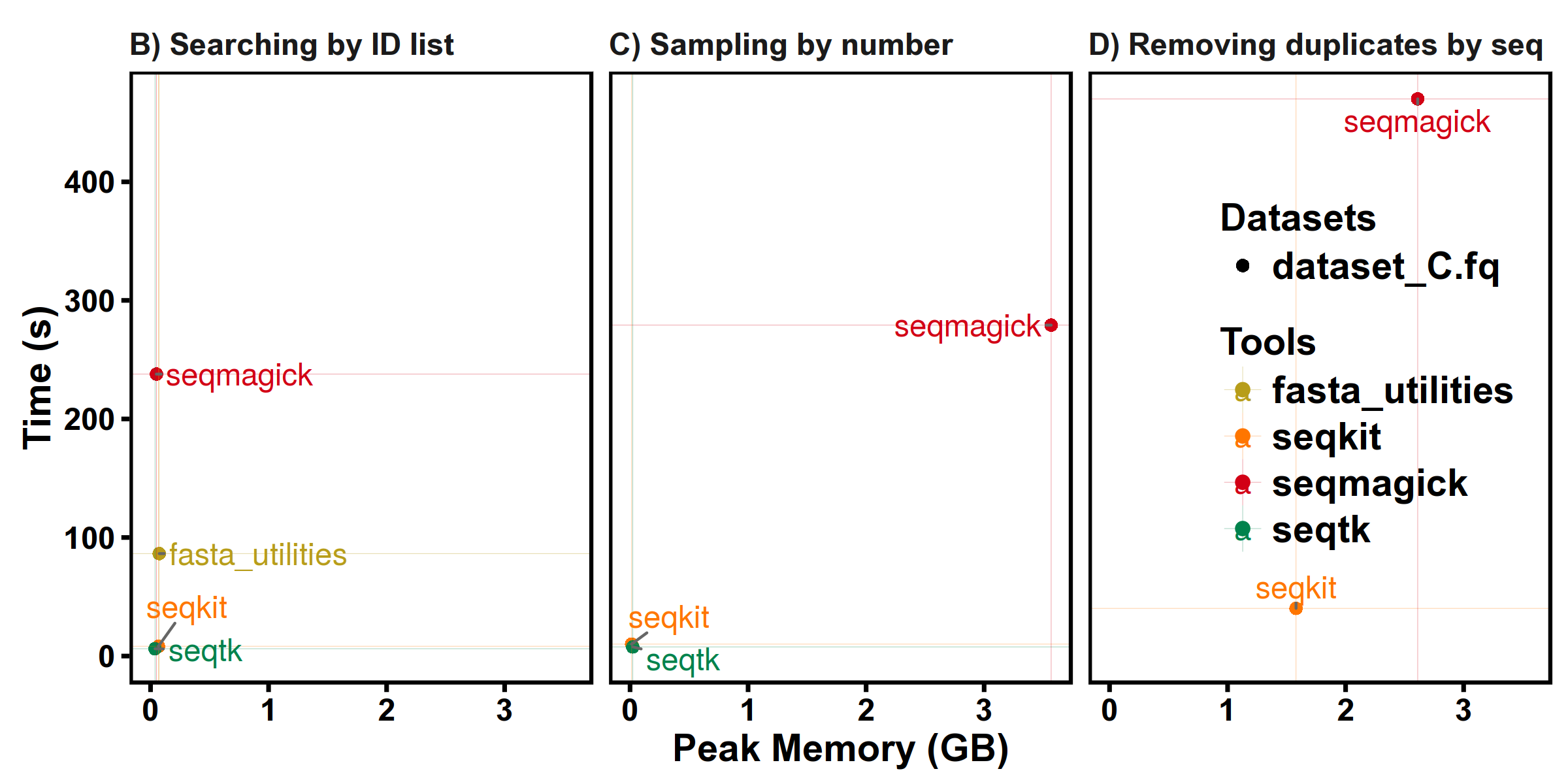

Supplement: S2 File — All data supporting this article including source code, documents, executable binary files, benchmark scripts and plotting scripts. (ZIP) [file pone.0163962.s002.zip › SeqKit-supplementary-data2/benchmark/benchmark.5tests.tsv.C.png]

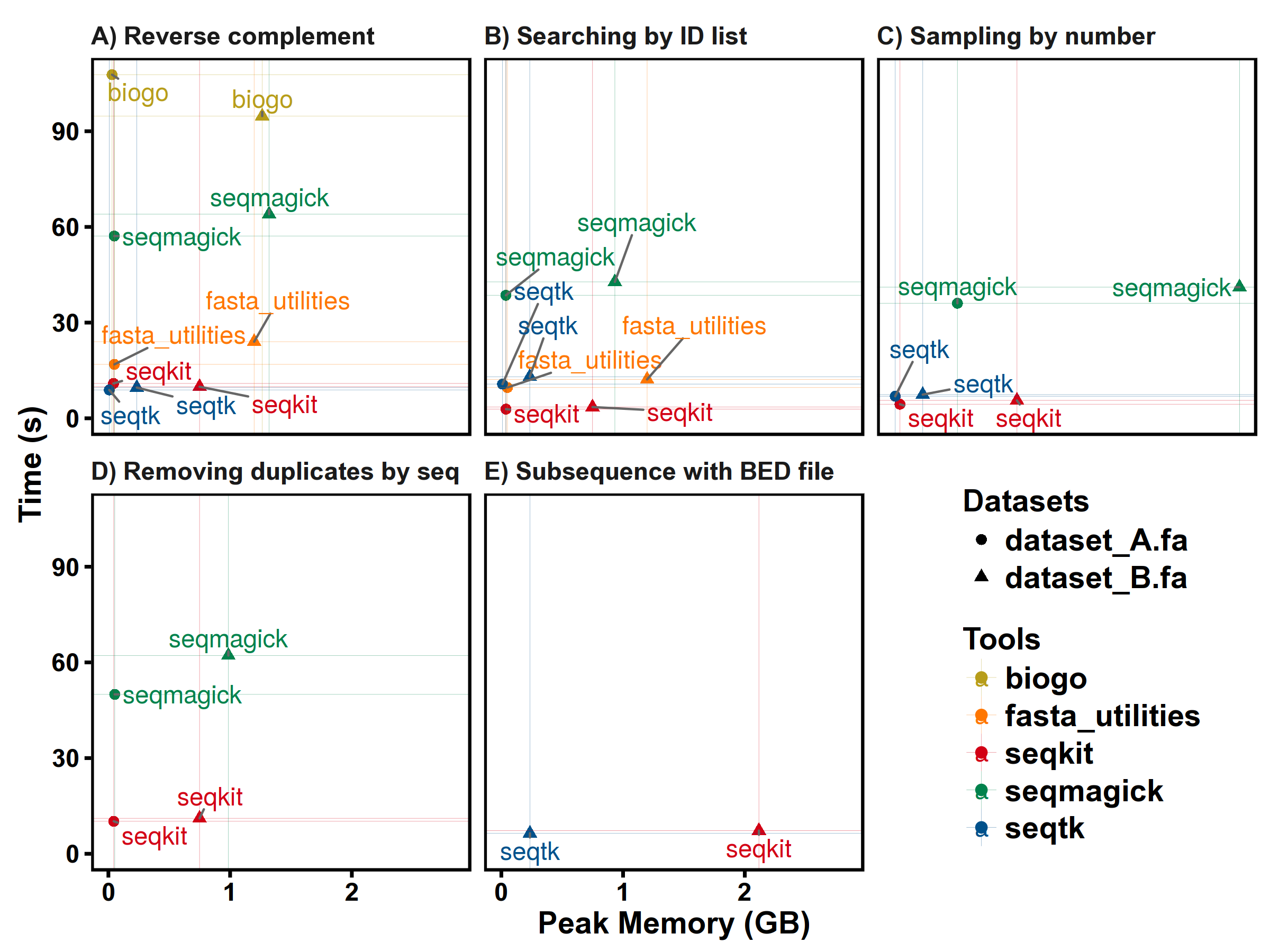

Supplement: S2 File — All data supporting this article including source code, documents, executable binary files, benchmark scripts and plotting scripts. (ZIP) [file pone.0163962.s002.zip › SeqKit-supplementary-data2/benchmark/benchmark.5tests.tsv.png]

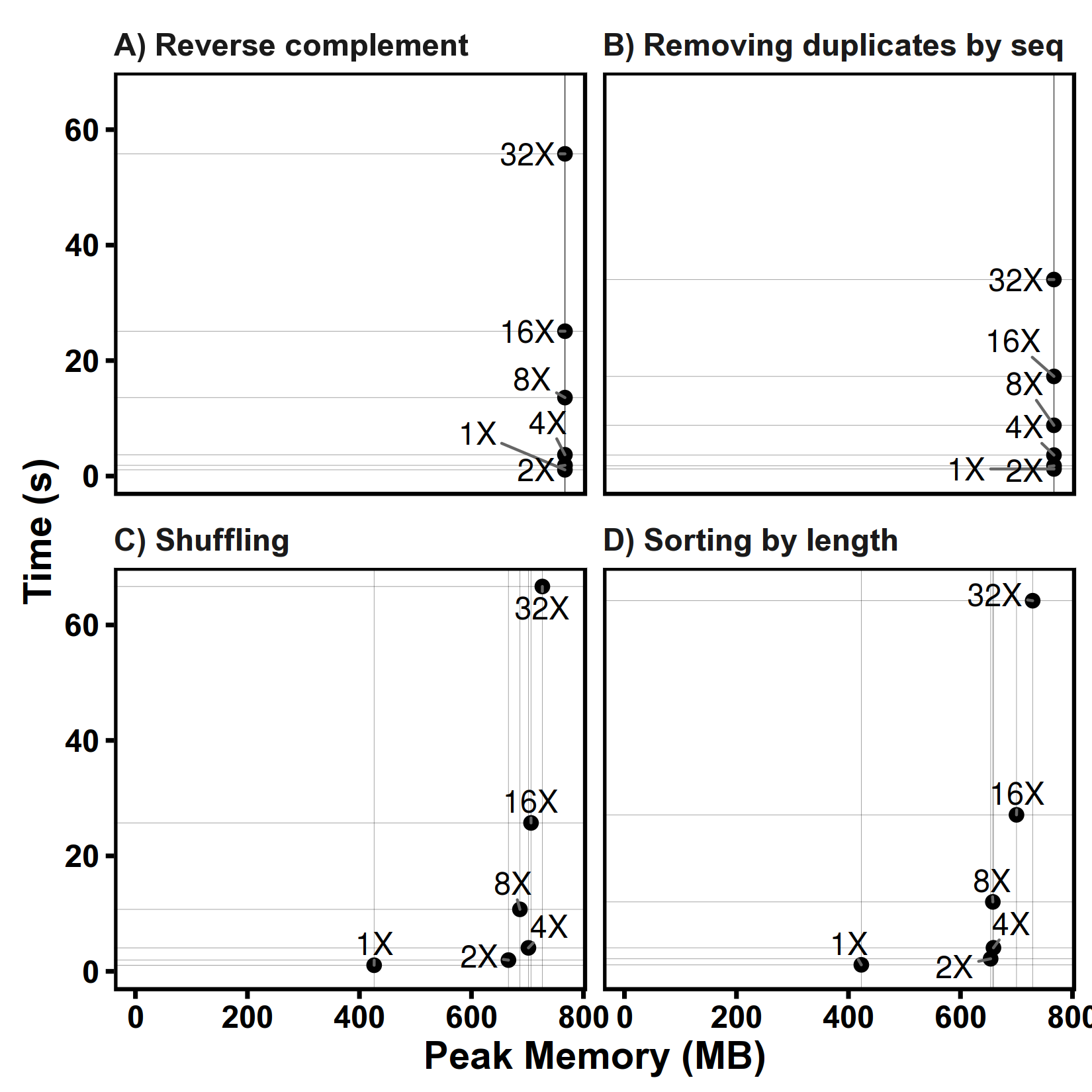

Supplement: S2 File — All data supporting this article including source code, documents, executable binary files, benchmark scripts and plotting scripts. (ZIP) [file pone.0163962.s002.zip › SeqKit-supplementary-data2/benchmark/seqkit_file_size/benchmark.seqkit.files_size.tsv.png]

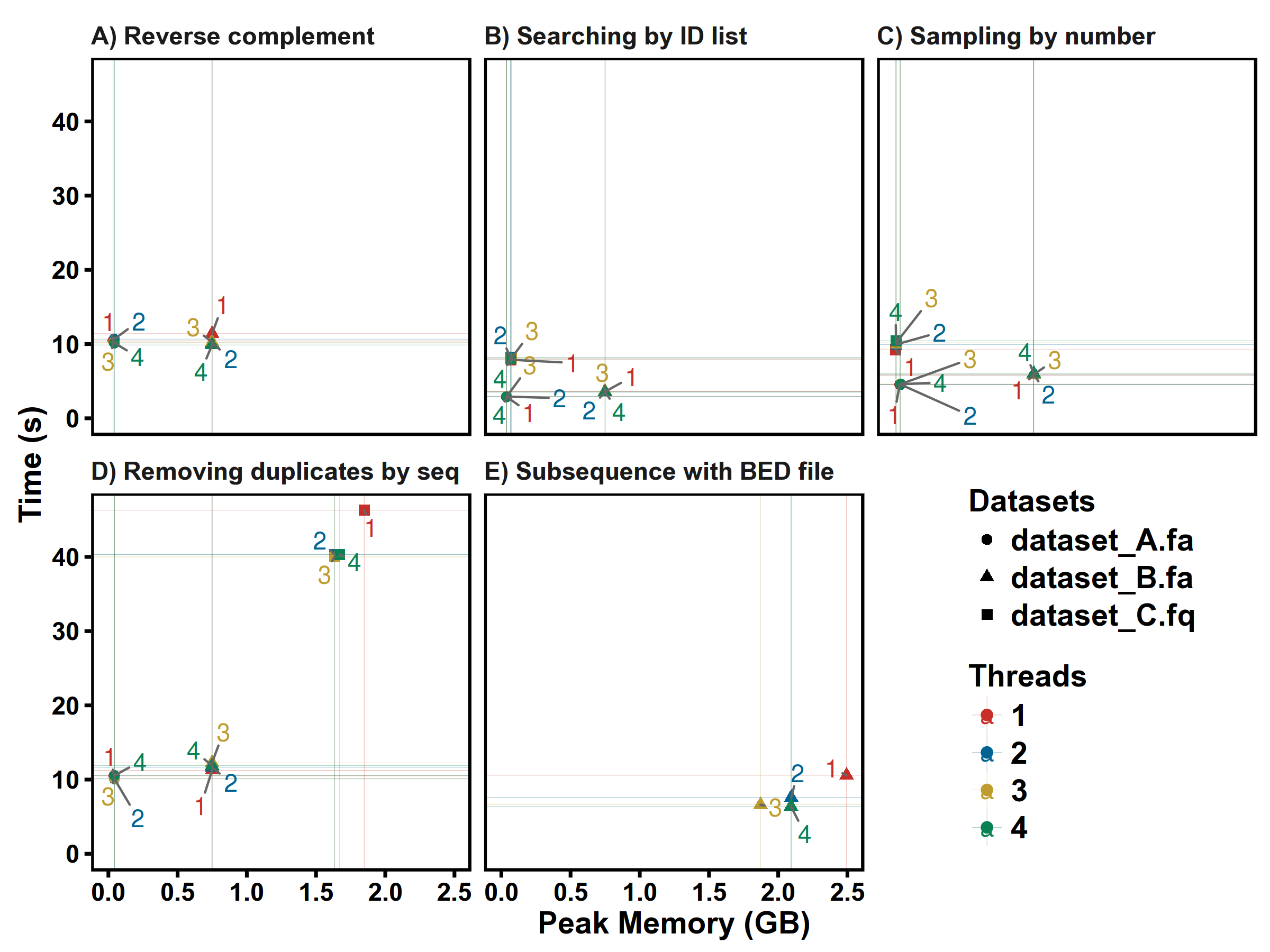

Supplement: S2 File — All data supporting this article including source code, documents, executable binary files, benchmark scripts and plotting scripts. (ZIP) [file pone.0163962.s002.zip › SeqKit-supplementary-data2/benchmark/seqkit_multi_threads/benchmark.5tests.tsv.png]

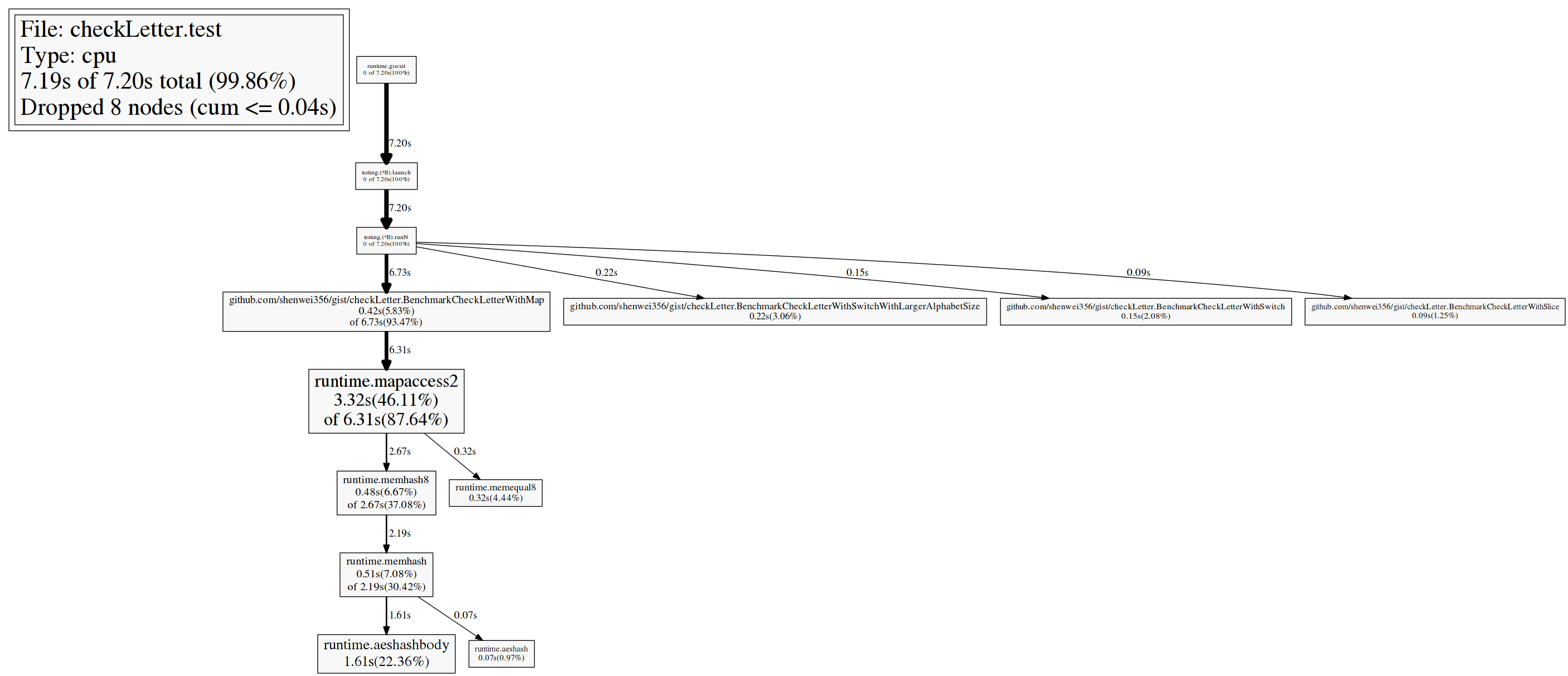

Supplement: S2 File — All data supporting this article including source code, documents, executable binary files, benchmark scripts and plotting scripts. (ZIP) [file pone.0163962.s002.zip › SeqKit-supplementary-data2/checkLetter/pprof.png]

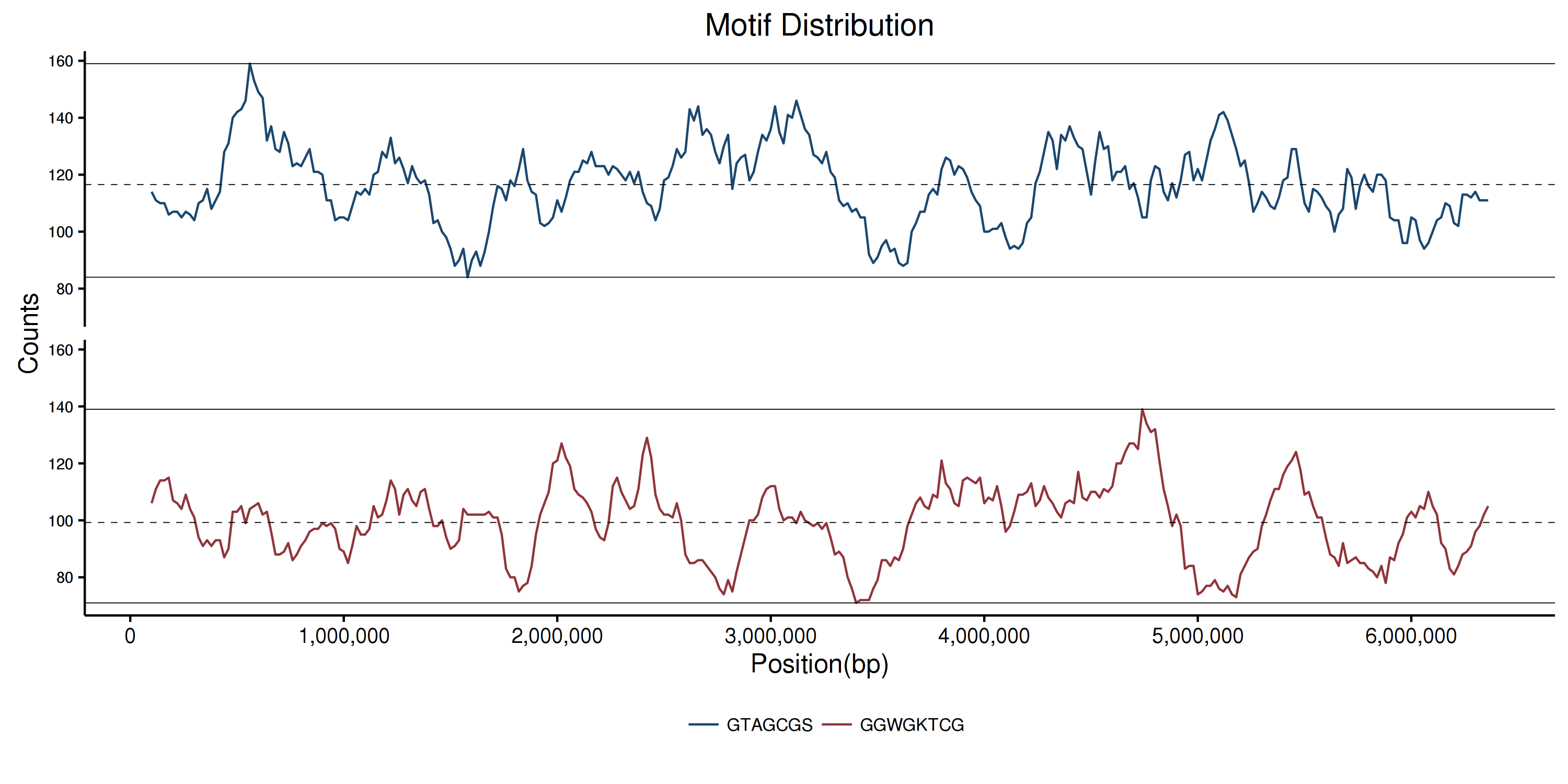

Supplement: S2 File — All data supporting this article including source code, documents, executable binary files, benchmark scripts and plotting scripts. (ZIP) [file pone.0163962.s002.zip › SeqKit-supplementary-data2/doc/docs/files/PAO1/motif_distribution.png]

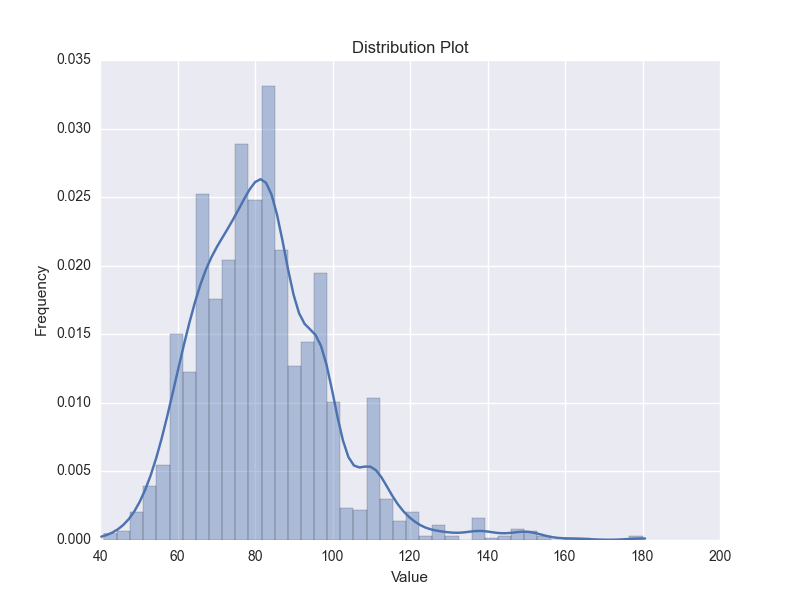

Supplement: S2 File — All data supporting this article including source code, documents, executable binary files, benchmark scripts and plotting scripts. (ZIP) [file pone.0163962.s002.zip › SeqKit-supplementary-data2/doc/docs/files/hairpin/hairpin.id_hsa.fa.gz.lendist.png]

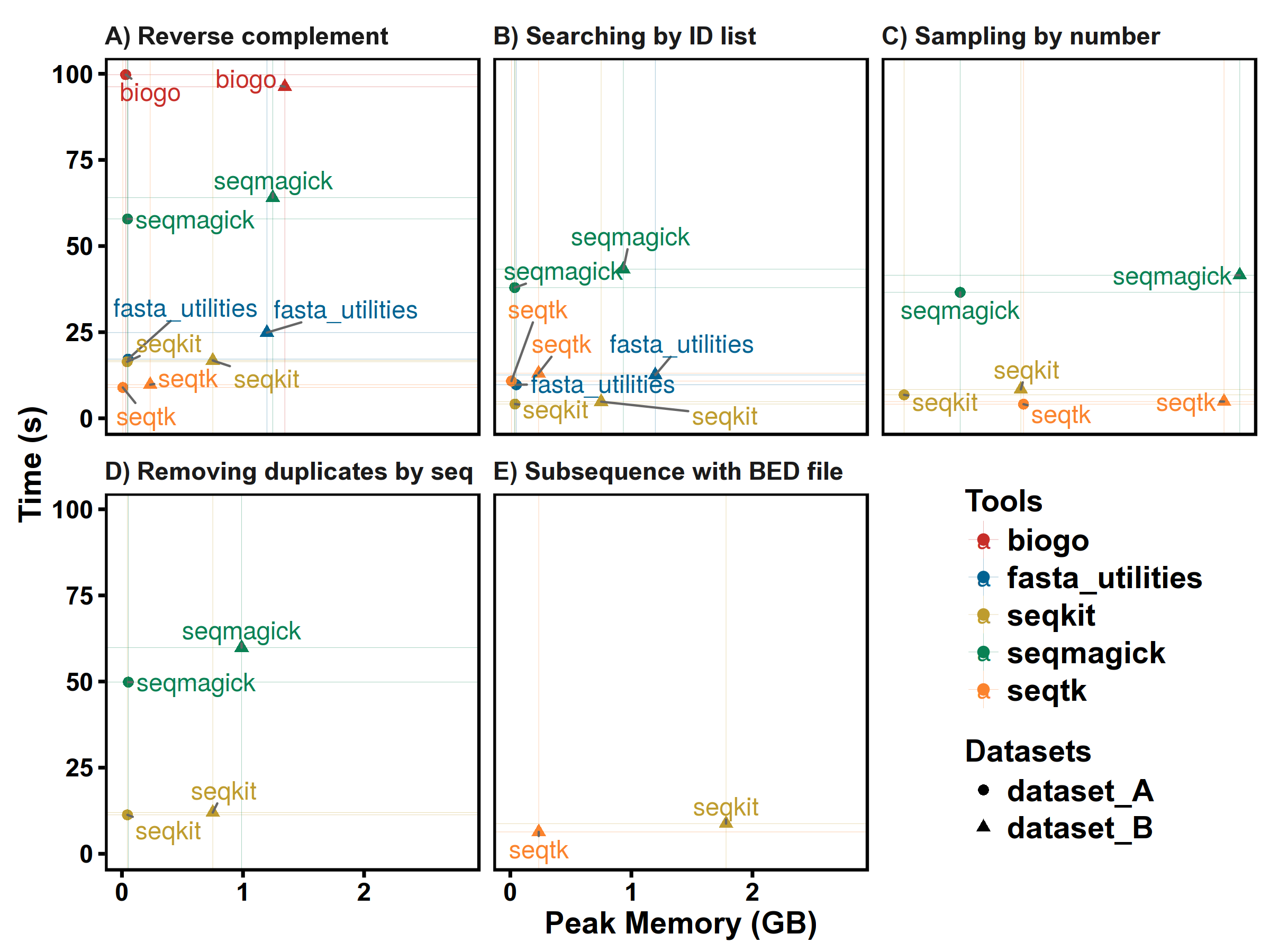

Supplement: S2 File — All data supporting this article including source code, documents, executable binary files, benchmark scripts and plotting scripts. (ZIP) [file pone.0163962.s002.zip › SeqKit-supplementary-data2/doc/site/benchmark/benchmark.5tests.csv.png]

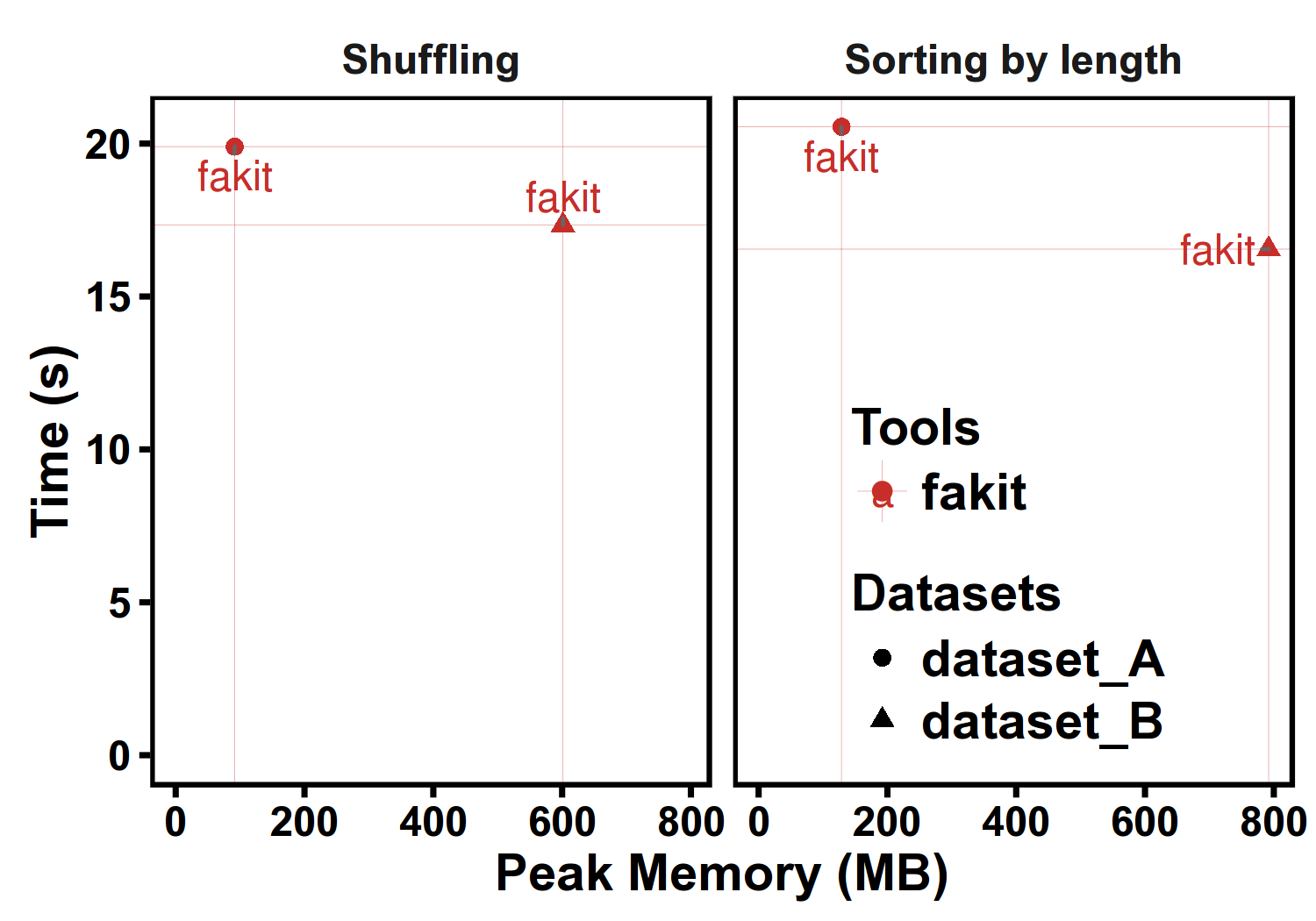

Supplement: S2 File — All data supporting this article including source code, documents, executable binary files, benchmark scripts and plotting scripts. (ZIP) [file pone.0163962.s002.zip › SeqKit-supplementary-data2/doc/site/benchmark/benchmark.seqkit.csv.png]

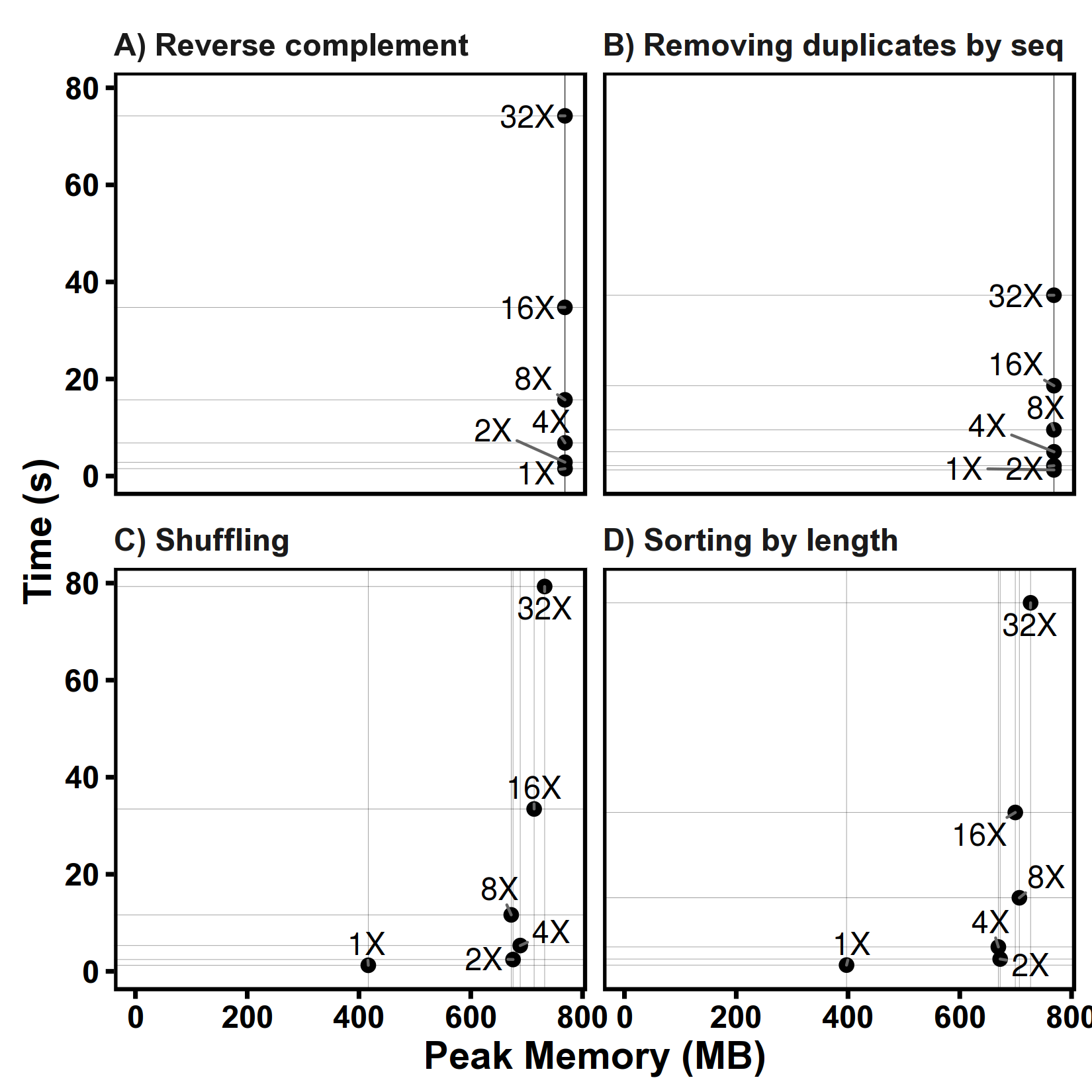

Supplement: S2 File — All data supporting this article including source code, documents, executable binary files, benchmark scripts and plotting scripts. (ZIP) [file pone.0163962.s002.zip › SeqKit-supplementary-data2/doc/site/benchmark/seqkit_file_size/benchmark.seqkit.files_size.csv.png]

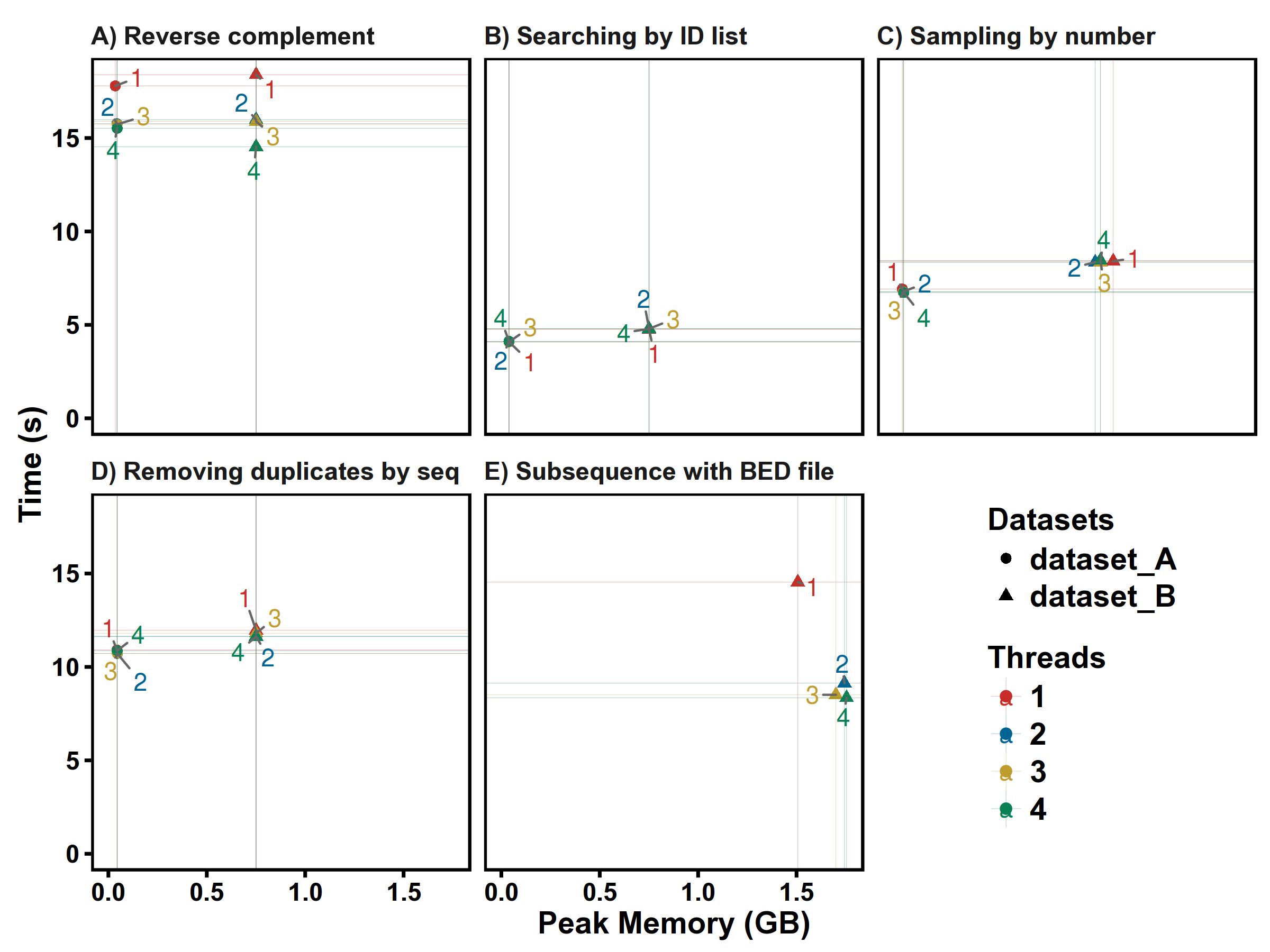

Supplement: S2 File — All data supporting this article including source code, documents, executable binary files, benchmark scripts and plotting scripts. (ZIP) [file pone.0163962.s002.zip › SeqKit-supplementary-data2/doc/site/benchmark/seqkit_multi_threads/benchmark.5tests.csv.png]

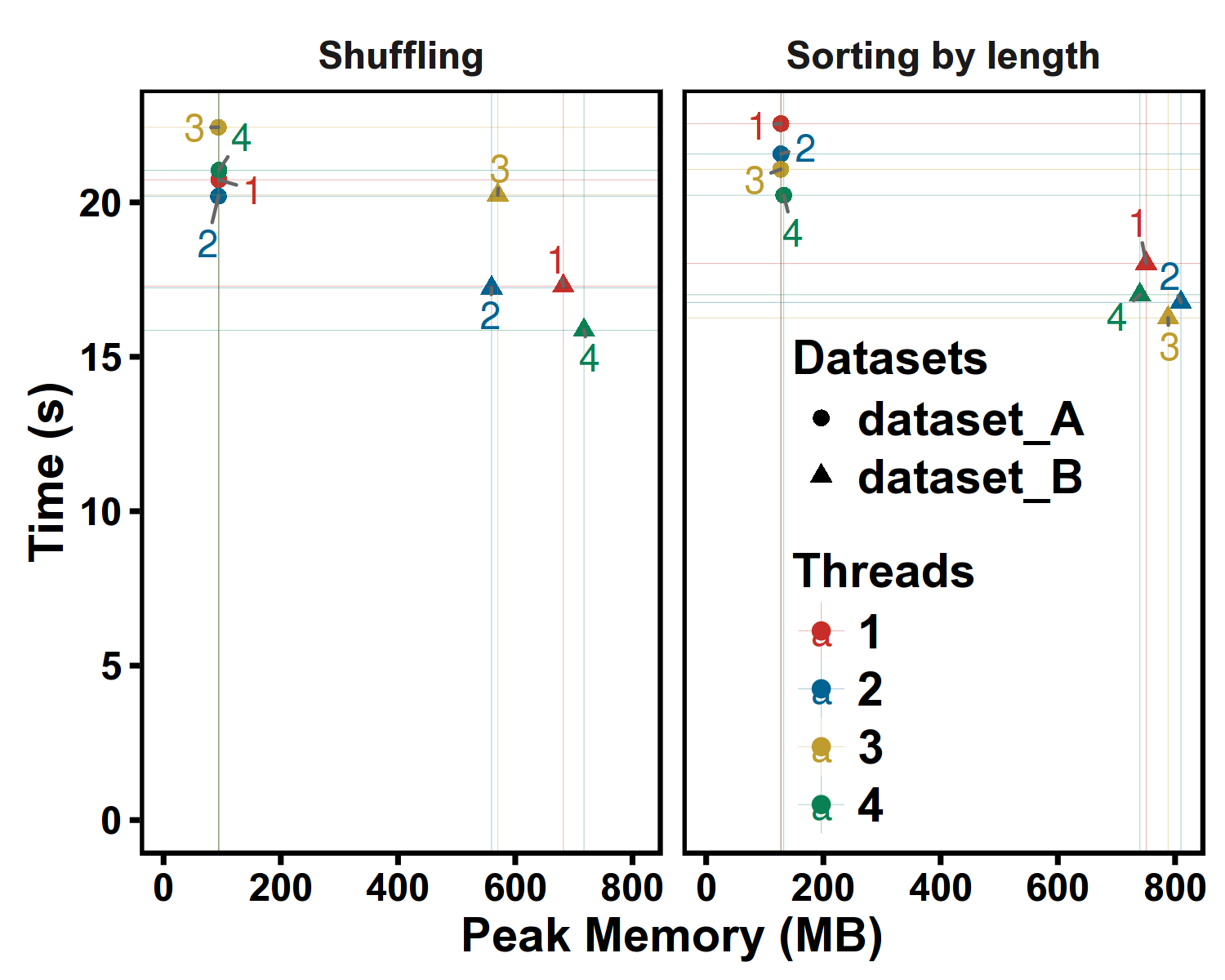

Supplement: S2 File — All data supporting this article including source code, documents, executable binary files, benchmark scripts and plotting scripts. (ZIP) [file pone.0163962.s002.zip › SeqKit-supplementary-data2/doc/site/benchmark/seqkit_multi_threads/benchmark.seqkit.csv.png]

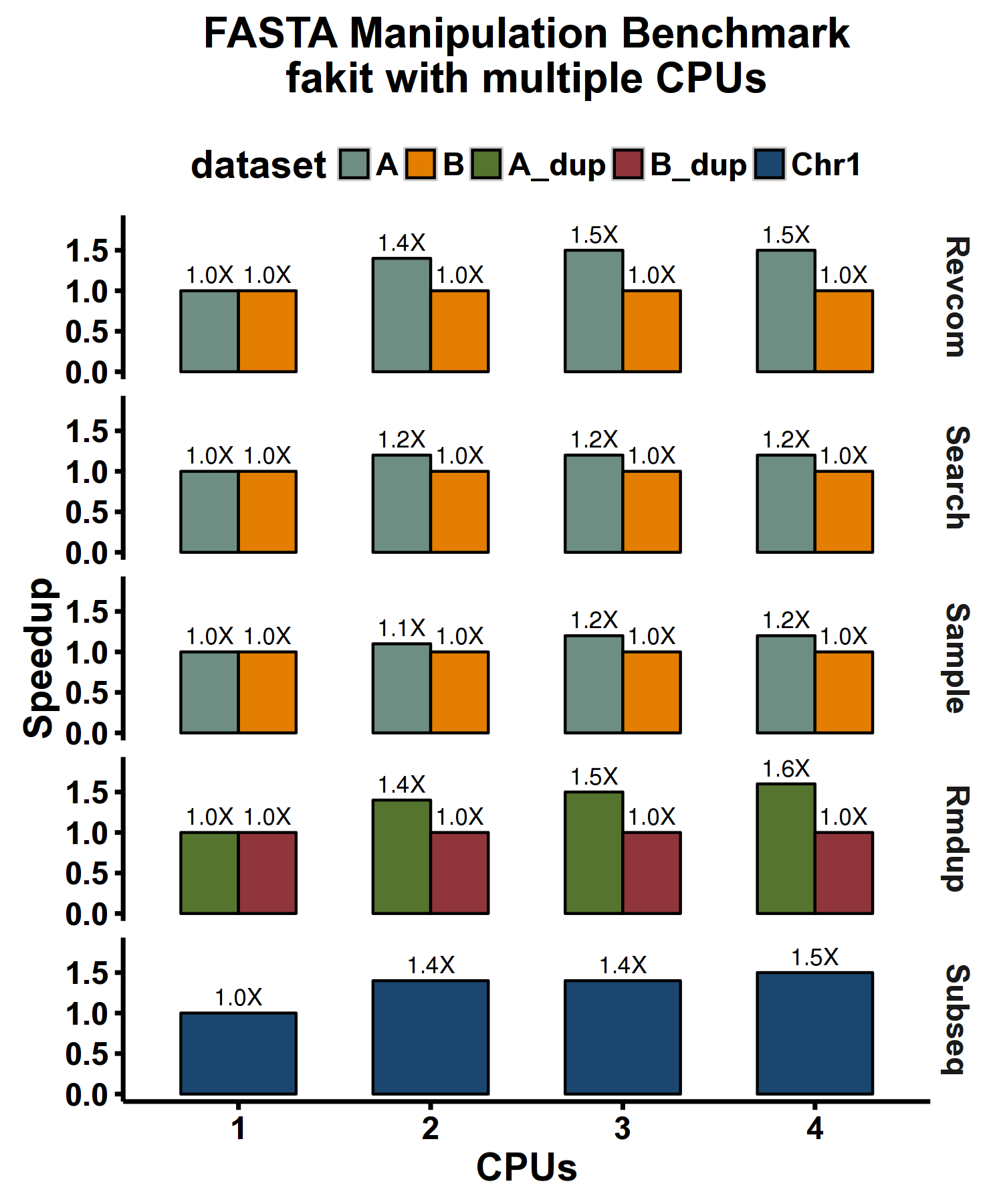

Supplement: S2 File — All data supporting this article including source code, documents, executable binary files, benchmark scripts and plotting scripts. (ZIP) [file pone.0163962.s002.zip › SeqKit-supplementary-data2/doc/site/benchmark/seqkit_multi_threads/benchmark_colorful.png]

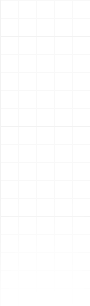

Supplement: S2 File — All data supporting this article including source code, documents, executable binary files, benchmark scripts and plotting scripts. (ZIP) [file pone.0163962.s002.zip › SeqKit-supplementary-data2/doc/site/img/grid.png]
